# Louse flies of Eleonora’s falcons that also feed on their prey are evolutionary dead‐end hosts for blood parasites

**DOI:** 10.1111/mec.15020

**Published:** 2019-04-04

**Authors:** Laura Gangoso, Rafael Gutiérrez‐López, Josué Martínez‐de la Puente, Jordi Figuerola

**Affiliations:** ^1^ Institute for Biodiversity and Ecosystem Dynamics (IBED) University of Amsterdam Amsterdam The Netherlands; ^2^ Department of Wetland Ecology Estación Biológica de Doñana (EBD‐CSIC) Seville Spain; ^3^ Centro de Investigación Biomédica en Red de Epidemiología y Salud Pública (CIBERESP) Madrid Spain

**Keywords:** avian malaria, coevolution, *Haemoproteus*, host switch, insular ecosystem, *Plasmodium*, vector switch

## Abstract

Host shifts are widespread among avian haemosporidians, although the success of transmission depends upon parasite‐host and parasite‐vector compatibility. Insular avifaunas are typically characterized by a low prevalence and diversity of haemosporidians, although the underlying ecological and evolutionary processes remain unclear. We investigated the parasite transmission network in an insular system formed by Eleonora's falcons (the avian host), louse flies that parasitize the falcons (the potential vector), and haemosporidians (the parasites). We found a great diversity of parasites in louse flies (16 *Haemoproteus* and 6 *Plasmodium* lineages) that did not match with lineages previously found infecting adult falcons (only one shared lineage). Because Eleonora's falcon feeds on migratory passerines hunted over the ocean, we sampled falcon kills in search of the origin of parasites found in louse flies. Surprisingly, louse flies shared 10 of the 18 different parasite lineages infecting falcon kills. Phylogenetic analyses revealed that all lineages found in louse flies (including five new lineages) corresponded to *Haemoproteus* and *Plasmodium* parasites infecting Passeriformes. We found molecular evidence of louse flies feeding on passerines hunted by falcons. The lack of infection in nestlings and the mismatch between the lineages isolated in adult falcons and louse flies suggest that despite louse flies’ contact with a diverse array of parasites, no successful transmission to Eleonora's falcon occurs. This could be due to the falcons’ resistance to infection, the inability of parasites to develop in these phylogenetically distant species, or the inability of haemosporidian lineages to complete their development in louse flies.

## 1 INTRODUCTION

Host shifts are widespread across parasite taxa, although the factors that determine the success or failure of these events are complex and strongly dependent on ecological (e.g., climate, geographical, or vector‐imposed barriers) and finely tuned parasite/host‐related processes, such as host specificity and parasite adaptability, as well as host immune mechanisms (Gager, Del Rosario Loaiza, Dearborn, & Bermingham, 2008; Lee et al., 2017; Moens et al., 2016; Sieber & Gudelj, 2014).

The biting behaviour of blood‐feeding arthropods can largely determine host‐parasite contact rates and consequently the transmission networks of vector‐borne parasites (Martínez‐de la Puente et al., 2015; Takken & Verhulst, 2013; Yan, Gangoso, Martínez‐de la Puente, Soriguer, & Figuerola, 2017). Blood parasites infecting a particular host may interact with a diversity of blood‐feeding arthropods that are competent or refractory for the transmission of the pathogen. Despite having similar life cycles, avian malarial parasites of the genus *Plasmodium* and the phylogenetically related *Haemoproteus* are transmitted by different dipterian insect vectors. While mosquitoes (Culicidae) transmit *Plasmodium*, *Culicoides* (Ceratopogonidae) and louse flies (Hippoboscidae) are the main vectors of *Haemoproteus* parasites of the subgenera *Parahaemoproteus* and *Haemoproteus*, respectively (Valkiūnas, 2005). However, the insect vectors of these avian parasites exhibit relatively opportunistic behaviour by feeding on the blood of different bird species, which could lead to host switching. Indeed, host shifts are frequent, rapid processes that have shaped the evolutionary history of avian haemosporidians (Alcala, Jenkins, Christe, & Vuilleumier, 2017; Ricklefs et al., 2014). Bird‐parasite interactions have been intensively studied in order to identify specificity among avian haemosporidians and different hosts (Clark, Clegg, & Lima, 2014; Križanauskienė et al., 2006; Palinauskas, Valkiūnas, Bolshakov, & Bensch, 2008; Valkiūnas, 2005); however, very little attention has been paid to the occurrence of vector shifts, and, in general, to the role of vector feeding behaviour in facilitating or inhibiting host shifts (Gager et al., 2008; Ishtiaq et al., 2008; Kim & Tsuda, 2010). This is partly due to the fact that, although the research on haemosporidian vectors has recently exploded, information on the vector breadth of the astounding diversity of within‐genera avian malarial lineages is still limited (Bobeva, Zehtindjiev, Bensch, & Radrova, 2013; Synek, Munclinger, Albrecht, & Votýpka, 2013; Valkiūnas, 2005).

Environmental conditions and their effects on vector populations strongly affect the transmission dynamics of avian haemosporidians (Ferraguti et al., 2018). On oceanic islands, where populations of insect vectors are usually limited by the prevailing conditions of high wind speeds and salinity, louse flies may play a key role in haematozoan transmission. For example, frigatebirds are commonly infected by *Haemoproteus* parasites including *Haemoproteus iwa,* which are vectored by louse flies (Levin et al., 2011; Merino et al., 2012). Similarly, *Haemoproteus multipigmentatus *infecting endemic Galápagos doves (*Zenaida galapagoensis*) is transmitted by the louse fly *Microlynchia galapagoensis* (Valkiūnas, Santiago‐Alarcon, Levin, Iezhova, & Parker, 2010). In an insular ecosystem, the opportunities for parasite spillover and diversification can be limited because of the low number of interacting species and low habitat diversity. However, vacant niches, in the form of new vectors and hosts, are available for newly arriving generalist strategists that are capable of exploiting new opportunities, thus broadening host ranges, but also for parasites that are able to flourish in new host‐vector assemblages, which in this case would promote parasite diversification (Agosta & Klemens, 2008; Drovetski et al., 2014; Medeiros, Ellis, & Ricklefs, 2014; Santiago‐Alarcon, Rodríguez‐Ferraro, Parker, & Ricklefs, 2014). In this context, the fact that louse flies are able to move between host individuals of the same or even different species, potentially increases the probability of host switching by haemosporidians (Jaramillo, Rohrer, & Parker, 2017; Levin & Parker, 2014). This may be the case for *H. multipigmentatus*, in that louse flies could have facilitated parasites jumping from doves to distantly related avian hosts on oceanic islands (Jaramillo et al., 2017; Levin & Parker, 2014; Levin et al., 2011).

Here, we investigated the parasite transmission network in an insular system formed by falcons (the avian host), louse flies that parasitize the falcons (the potential vector), and avian haemosporidians (the parasites). The Eleonora's falcon (*Falco eleonorae*) is a medium‐sized long‐distance migratory raptor that breeds on islands in the Mediterranean basin and winters in Madagascar (Kassara et al., 2017; Walter, 1979). Adult Eleonora's falcons are commonly infected by *Plasmodium* and *Haemoproteus* parasites (Gangoso, Gutiérrez‐López, Martínez‐de la Puente, & Figuerola, 2016; Gutiérrez‐López, Gangoso et al., 2015a). In addition, both adults and nestlings are heavily parasitized by the louse fly *Ornithophila gestroi* (Gangoso et al., 2010), which has only been reported on Eleonora's falcon and the closely related common and lesser kestrels (*Falco tinnunculus and Falco naumanni*) (Beaucournu, Beaucournu‐Saguez, & Guiguen, 1985; Gangoso et al., 2010; Walter, 1979). Louse flies may play a critical role in the transmission dynamics of blood parasites in marine ecosystems, as has been found for different *Haemoproteus* lineages (Levin, Valkiūnas, Iezhova, O'Brien, & Parker, 2012; Levin et al., 2011; Valkiūnas et al., 2010). We therefore hypothesized that Eleonora's falcons and louse flies would share the same and rather small number of haemosporidian lineages already reported in the Eleonora's falcon. However, we found a completely different scenario. Louse flies were infected by an unexpectedly high diversity of parasite lineages, all of them typical of passeriform birds. Therefore, we investigated the origin of these haemosporidians infecting louse flies. During the breeding season, Eleonora's falcons prey on European migratory passerines intercepted over the ocean while heading to Africa, and the dead birds are stored in larders around the falcons’ nest sites (Viana, Gangoso, Bouten, & Figuerola, 2016). Taking advantage of this behaviour, we sampled the falcon kills and included this new piece in the host‐vector‐parasite system studied.

## 2 MATERIALS AND METHODS

We sampled louse flies in September 2011–2013 on Alegranza islet (Canary Islands; 1,050 ha, 289 m above sea level). Nestling Eleonora's falcons that were 25–28 days old were inspected for louse flies for 5 min by focusing on the pericloacal area, where these insects usually concentrate (authors’ personal observation). Louse flies (range 1–9) were removed from each individual bird and immediately immersed in absolute ethanol in 2011 (159 louse flies sampled from 50 nests). In 2012 and 2013, louse flies were kept alive for four days in empty plastic containers before being placed in Eppendorf tubes filled with absolute ethanol (369 louse flies from 64 nests in 2012 and 499 louse flies from 63 nests in 2013). During that period the louse flies digested any vertebrate blood present in their abdomen reducing the potential to detect parasites only present in the blood meal and not in the insect tissues. Host DNA is usually degraded after a few days post‐ingestion (Martínez‐de la Puente, Ruiz, Soriguer, & Figuerola, 2013). In fact, host DNA was undetectable in mosquito vectors tested 72 hr after blood feeding (Hiroshige et al., 2017). The single louse fly species that was collected from the study area corresponded to *O. gestroi*, according to morphological and genetic characterization of the specimens (Gutiérrez‐López, Martínez‐de la Puente, Gangoso, Soriguer, & Figuerola, 2015b).

To identify the avian hosts of the blood parasites that were isolated from the louse flies, blood samples from both adult and nestling Eleonora's falcons and bird prey were obtained. Blood parasites infecting Eleonora´s falcons that were sampled from 2006 to 2014 (*N* = 173 nestlings and 209 samples from 183 adults) had been previously analyzed (Gangoso et al., 2016; Gutiérrez‐López, Gangoso et al., 2015a). In addition, in September 2013 we sampled 90 recent kills of 12 bird species belonging to seven different families. We obtained a fresh blood sample (*N* = 14) or heart tissue with blood that was nearly coagulated (*N* = 76) from each bird, which were immediately stored in Eppendorf tubes filled with absolute ethanol. We left the sampled kill in the same place it was found for later consumption by the falcons. All of the samples were stored at –20ºC until molecular analyses were performed.

### 2.1 Molecular analysis

Genomic DNA was extracted from whole louse flies collected in 2011 using a common chloroform/isoamyl alcohol protocol. For the louse flies collected in 2012–2013, the head‐thorax of each fly was separated from the abdomen using sterile scalpel blades and forceps on sterile Petri dishes and each part stored separately in individual tubes with absolute ethanol. The presence of parasite DNA in the head‐thorax may indicate that the parasite has developed in the insect, and potentially colonized the salivary glands, which are located in the head‐thorax, thus being potentially able to be transmitted to a new host. Nonetheless, positive amplifications in the head‐thorax, even in insects that have completely digested blood meals, do not guarantee that the parasite has completed its multiplicative cycle nor the vector competence of the insect, as parasite DNA could be amplified from abortive forms of the parasite (Santiago‐Alarcón, Palinauskas, & Schaefer, 2012; Valkiūnas, Kazlauskienė, Bernotienė, Palinauskas, & Iezhova, 2013). Genomic DNA from the head‐thorax was extracted using a MAXWELL® 16 LEV Blood DNA Kit (see Gutiérrez‐López, Martínez‐de la Puente et al., 2015b). The same procedure was used to extract the genomic DNA from blood samples or heart tissue from fresh bird kills. Fresh organs, including the heart, can be successfully used to identify the exoerythrocytic stages of avian malarial infections using molecular tools, although the prevalence of infection may be underestimated (Mendes et al., 2013).

We determined the presence and identity of *Haemoproteus* and *Plasmodium* parasites in the head‐thorax of louse flies and in bird kills following the method of Hellgren, Waldenström, and Bensch (2004). The presence of amplicons was verified in 1.8% agarose gels, and positive samples were sequenced using the BigDye^®^ technology (Applied Biosystems) or the Macrogen sequencing service (Macrogen Inc., Amsterdam, the Netherlands). Sequences were edited using the software Sequencher™ v 4.9 (Gene Codes Corp., Ann Arbor, MI) and quality of the reads was between 88.9% and 100% in samples from louse flies and between 80.7% and 99.3% in samples from bird kills, except two samples that showed qualities of 64% and 73%, respectively. Sequences were assigned to parasite lineages/morphospecies after comparison with the GenBank (National Centre for Biotechnology Information) and MalAvi (Bensch, Hellgren, & Pérez‐Tris, 2009) databases. For all the sequences that differed by at least one base from a lineage previously isolated (see Table S1 of the Supplemental Information), we amplified the DNA and sequenced the *cytb* gene twice from independent reactions to exclude the possibility that these putative new lineages were due to degraded DNA and/or sequencing errors. Sequences identified for the first time were deposited in GenBank (accession numbers: MH271173‐ MH271183).

The abdomens of 80 louse flies collected in 2012 with a positive amplification of parasites in the head‐thorax were further analyzed to determine whether the parasite identified could be due to the presence of any rest of an undigested blood meal and also to identify the potential presence of host DNA. We extracted genomic DNA from the abdomen of louse flies using the Maxwell‐based protocol described above and analyzed the presence of DNA from hosts and parasites following Alcaide et al. (2009) and Hellgren et al. (2004), respectively.

### 2.2 Statistical and phylogenetic analyses

For statistical analyses, we excluded results from 2011 because the protocol used for the manipulation of louse flies does not allow clarifying whether parasites really infected louse flies or are amplifications derived from an undigested blood meal. We also excluded parasite lineages identified from the louse flies abdomens (only 23.8% provided positive amplifications of parasite DNA). Ten louse flies and four bird kills showed evidence of coinfection by at least two haemosporidian lineages*, *based on the double peaks found in the chromatograms, so were not included in the statistical tests.

We assessed differences in the parasite prevalence in head‐thoraxes of louse flies between 2012 and 2013 by using a Generalized Linear Mixed Model (GLMM) with binomial error and logit link function in R v3.4.3 (R Core Team, 2017) with the package *lme4* (Bates, Maechler, Bolker, & Walker, 2015). The parasite infection status defined as a binary variable (0/1) was used as the response variable. We grouped information from the two parasite genera and did not fit a different model for *Plasmodium* and *Haemoproteus* due to the low prevalence of *Plasmodium *infections (2.44% and 1.40% in 2012 and 2013, respectively) found in louse flies. The nest identity where louse flies were collected was included as a random term. The similarity of lineages sequenced in the louse flies and migratory birds was evaluated using the Jaccard similarity index (Jaccard, 1902), which ranges from 0 (no similarity) to 1 (complete similarity). The statistical significance of the result was established using the critical value of Jaccard's similarity index at the 95% confidence level (Real, 1999).

We assessed the phylogenetic relationships of the 24 *Haemoproteus* and 10 *Plasmodium* lineages found in head‐thorax of louse flies, Eleonora's falcons and falcon kills with sequences from 69 *Haemoproteus* and 29 *Plasmodium *lineages of known morphospecies deposited in MalAvi (Bensch et al., 2009; accessed May 2018). Sequences were aligned using the CLUSTALW algorithm implemented in MEGA7 (Kumar, Stecher, & Tamura, 2016). We used 478 bp fragments to analyze the phylogenetic relationships between lineages using a maximum likelihood algorithm based on the Jukes‐Cantor model (Jukes & Cantor, 1969). Jukes‐Cantor model was selected as the best model based on the AIC criteria in JModeltest 2.1.10. Nodal support was estimated by bootstrap analysis with 1,000 replications (Felsenstein, 1981). We used the *Leucocytozoon* lineage L_CIAE02 as outgroup.

## 3 RESULTS

### 3.1 Louse flies

Overall, 22 different parasite lineages were identified in the head thorax of louse flies from 2012 and 2013 (16 *Haemoproteus* spp. and six *Plasmodium* spp.) including five new *Haemoproteus* lineages described here for the first time (lineages named as ORGES1, ORGES2, ORGES3, ORGES4 and ORGES5; GenBank accession numbers MH271176‐80) (Table 1, see also Table S1 of the Supplemental Information reporting pairwise distances between parasite lineages)*.* Two additional *Haemoproteus *parasites were also found in samples from 2011, but amplifications could only be identified to genus level (Table 1). The most common parasite lineage isolated from the louse flies was PFC1 (50.7% of total infections, *N* = 223), followed by HIPOL1 (22.9%). The remaining parasites were found in ≤7 louse flies (see Table 1). Parasite prevalence in head‐thoraxes of louse flies did not differ between 2012 and 2013 (year: estimate = 0.008 ± 0.27 standard error, *χ* ^2^ = 9e‐04, *df* = 1, *p* = 0.98, *N* = 785).

**Table 1:** Number of infected louse flies and falcon kills by the *Haemoproteus* and *Plasmodium* lineages found in this study as well as of adult Eleonora's falcons by previously found haemosporidian lineages (Gangoso et al., 2016; Gutiérrez‐López, Gangoso et al., 2015a)

	Louse flies			Falcon kills	Eleonora's falcons
Year	2011	2012	2013	2013	2006–2014
Total analyzed	159	369	499	90	209
Total positive	64	89	134	67	27
Parasite lineage					
*H. *sp*.* LANSEN1*	0	0	3	5	0
*H. *sp. ACDUM2	0	1	1	3	0
*H. *sp. HIPOL4*	1	2	5	2	0
*H. attenuatus *ROBIN1	1	0	0	3	0
*H. balmorali *COLL3	1	3	1	2	0
*H. balmorali *SFC1	0	2	0	0	0
*H*. sp. ERU−15H	1	1	7	0	0
*H. *sp. HIPOL1	20	15	36	12	0
*H. pallidus *PFC1	27	50	63	19	0
*H. palloris *WW1	0	2	4	0	0
*H. payevsyi *RW1	0	0	1	0	0
*H. *sp. RBS3	2	0	1	1	0
*H. *sp. ORGES1*	0	1	0	0	0
*H. *sp. ORGES2*	0	1	0	0	0
*H. *sp. ORGES3*	0	0	1	0	0
*H. *sp. ORGES4*	0	0	1	0	0
*H. *sp. ORGES5*	1	0	1	0	0
*H. *sp. HIPOL5*	0	0	0	2	0
*H. *sp. PHYBON1*	0	0	0	1	0
*H. *sp. PHYTRO1*	0	0	0	1	0
*H. *sp. SYCAN02*	0	0	0	1	0
*H. *sp. LK4	0	0	0	0	3
*H. sp. *BUBIBI01	0	0	0	0	6
*H*. sp. FALELE01	0	0	0	0	1
*H. *spp.	2	0	0	0	0
*P. *sp. AFTRU5	1	1	0	0	0
*P. *sp. LK6	0	2	0	0	15
*P. relictum *GRW11	0	1	1	1	0
*P. relictum *SGS1	1	2	4	6	0
*P. *sp. SYAT24	0	0	1	0	0
*P. vaughani *SYAT05	0	3	1	1	0
*P. *sp. COLL1	0	0	0	1	0
*P. *sp. GRW9	0	0	0	1	0
*P. *sp. MOALB1	0	0	0	1	0
*P_ACCTAC01*	0	0	0	0	1
*L. *sp. CIAE02	0	0	0	0	1
Coinfection	6	2	2	4	0

Note

Results of louse flies from 2011 correspond to whole insect extracts, while results from 2012 and 2013 correspond to head‐thoraxes. New lineages described in this study are indicated with an asterisk.

Of the 80 louse flies collected in 2012 with infected head‐thoraxes, 19 abdomens (23.8%) had positive amplifications of parasites corresponding to the *Haemoproteus *lineages PFC1 (11.3%), HIPOL1 (3.8%), WW1 (3.8%), SFC1 (1.3%), and LK4 (1.3%) and the *Plasmodium *lineage LK6 (2.5%). Six louse flies had parasite lineages in their abdomens that were different to those found in their head‐thoraxes (see Table 2).

**Table 2:** *Haemoproteus* and *Plasmodium* lineages found in the head‐thorax and abdomen analyzed separately in 80 louse flies from 2012 (total positive = 19)

Head‐thorax	Abdomen	*N*
*H.* sp. PFC1	*H.* sp. PFC1	8
*H.* sp. SFC1	*H.* sp. SFC1	1
*H.* sp. PFC1	*H. *sp. HIPOL1	2
*H.* sp. PFC1	*P. *sp. LK6	1
*H. *sp. HIPOL1	*H. *sp. HIPOL1	1
*H. palloris *WW1	*H. palloris *WW1	2
*P. relictum *SGS1	*H.* sp. PFC1	1
*P. *sp. AFTRU5	*H. *sp. LK4	1
*P. *sp. LK6	*P. *sp. LK6	1
Coinfection	*H.* sp. PFC1	1

Note

“*N*” denotes the number of louse flies where the same parasite combination was found.

### 3.2 Falcon kills

Overall, 74.4% (*N* = 90) of Eleonora´s falcon kills were infected by parasites corresponding to six lineages of *Plasmodium *spp. and 12 lineages of *Haemoproteus* spp. (see Tables 1 and 3). These include six newly described lineages (i.e., HIPOL4 (GenBank reference MH271173) and HIPOL5 (MH271175) isolated from the melodious warbler (*Hippolais polyglotta*), LANSEN1 (MH271174) isolated from the woodchat shrike (*Lanius senator*), PHYBON1 (MH271181) isolated from the Western Bonelli's warbler (*Phylloscopus bonelli*), PHYTRO1 (MH271182) isolated from the willow warbler (*P. trochilus*) and SYCAN02 (MH271183) isolated from the subalpine warbler (*Sylvia cantillans*), see Table S1 of the Supplemental Information). The bird species that were recorded most frequently as prey across years were the European pied flycatcher (*Ficedula hypoleuca*) and the common whitethroat (*Sylvia communis*), while the frequency of other species differed over time (Table 3). European pied flycatchers were recorded in all nests in which positive louse flies were found, while common whitethroats were found in 35 of 38 nests. The most prevalent parasite lineages that were isolated from passerines were the same, common lineages found in louse flies, i.e., PFC1 (28.4% of total infections, *N* = 67) and HIPOL1 (17.9%). The remaining parasite lineages were found in ≤ 6 birds (see Table 1).

**Table 3:** Bird species hunted by Eleonora's falcons (falcon kills) during the study period and their relative frequency in the year of sampling (2013)

Bird species	2011 (57)	2012 (43)	2013 (99)	Frequency 2013	*N *sampled	Parasite lineages and prevalence within each bird species
*Acrocephalus paludicola*			2	0.002	2	*H. pallidus *PFC1 (0.5), *P*. sp.COLL1 (0.5)
*Acrocephalus scirpaceus*	2			0		
*Actitis hypoleucos*	7	4	44	0.05		
*Anthus trivialis*	1	1		0		
*Apus *spp.	1		18	0.02		
*Bulweria bulwerii*	1		32	0.04		
*Cercotrichas galactotes*	1			0		
*Clamator glandarius*			1	0.001		
*Coturnix coturnix*	10	12	34	0.04	2	0
*Crex crex*			7	0.008		
*Cuculus canorus*	3		9	0.01		
*Ficedula hypoleuca*	73	118	177	0.21	27	*H. pallidus *PFC1 (0.48),*H*. sp.LANSEN1 (0.04), *H. balmorali *COLL3 (0.07), *H*. sp. HIPOL1 (0.07), *P*. sp. GRW9 (0.04)
*Hippolais polyglotta*	33	44	59	0.07	16	*H*. sp. ACDUM2 (0.12), *H*. sp. HIPOL1 (0.5), *H*. sp. HIPOL4 (0.12), *H*. sp. HIPOL5 (0.06)
*Hippolais *spp.		3		0		
*Hydrobates pelagicus*	2		14	0.02		
*Jynx torquilla*	2	5	14	0.02		
*Lanius senator*	22	14	67	0.08	9	*H. sp. *LANSEN1 (0.44), *H. attenuatus *ROBIN1 (0.11), *H. sp. *RBS3 (0.11), *P. relictum *SGS1 (0.11)
*Locustella naevia*	21	3	7	0.008		
*Luscinia megarhynchos*	60	7	49	0.06	2	*H. attenuatus *ROBIN1 (1)
*Motacilla flava*		1	8	0.009	1	*P. sp. *MOALB1 (1)
*Muscicapa striata*	8	3		0		
*Oenanthe hispanica*			5	0.006		
*Oenanthe oenanthe*			38	0.05		
*Phalaropus fulicarius*		2	4	0.005		
*Phoenicurus ochruros*			34	0.04		
*Phoenicurus phoenicurus*	6	4	3	0.004	1	0
*Phylloscopus bonelli*	4	4	3	0.004	5	*H. sp. *HIPOL5 (0.2), *H. sp. *PHYBON1 (0.2), *P. relictum *SGS1 (0.2)
*Phylloscopus collybita*	8		4	0.005		
*Phylloscopus *spp*.*	13	11	39	0.05		
*Phylloscopus trochilus*	5	4	3	0.004	2	*H. pallidus *PFC1 (0.5), *H. sp. *PHYTRO1 (0.5)
*Streptopelia turtur*	2		12	0.01		
*Saxicola rubetra*		1		0		
*Sylvia cantillans*			2	0.002	2	*H. pallidus *PFC1 (0.5), *H. sp. *SYCAN02 (0.5)
*Sylvia communis*	70	47	139	0.17	21	*H. pallidus *PFC1 (0.14), *H. sp. *ACDUM2 (0.05), *H. sp. *HIPOL1 (0.09),* P. relictum *GRW11 (0.05), *P. relictum *SGS1 (0.2), *P. vaughani *SYAT05 (0.05)
*Sylvia *spp.	1	1		0		
*Upupa epops*	3	3	1	0.001		

Note

The number of nests sampled each year is indicated just below the year in parentheses. The number of individuals of each species sampled and the prevalence of different parasite lineages isolated within each species (next to each parasite lineage, in parentheses) are also shown.

### 3.3 Similarity between parasite faunas and possible origin of haemosporidians

**Figure 1:**
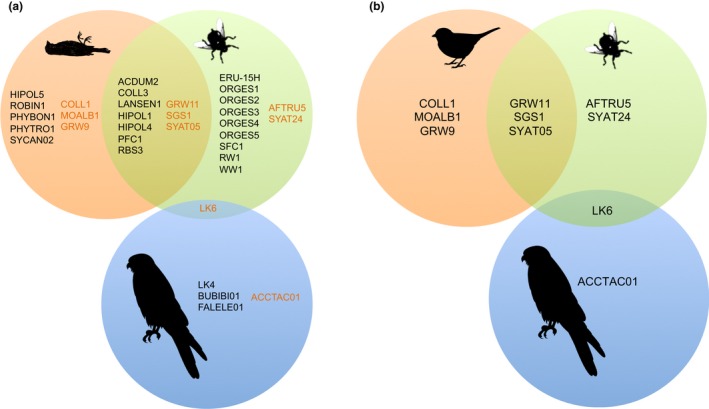
Lineage sharing (overlapped areas) of *Haemoproteus *(indicated with black letters) and *Plasmodium *(dark orange letters) parasites between falcon kills (orange sphere), louse flies (green sphere) and adult Eleonora's falcons (blue sphere). Lineages of louse flies (head‐thorax samples from 2012 to 2013) and falcon kills were identified in this study, while those of Eleonora's falcons were reported in Gangoso et al. (2016) and Gutiérrez‐López, Gangoso et al. (2015a)

Louse flies and falcon kills shared 10 parasite lineages (seven *Haemoproteus* and three *Plasmodium* lineages, see Figure 1). Their parasite faunas were moderately but not significantly similar, both when considering only parasite lineages isolated from louse flies collected in 2013, when kills were sampled (Jaccard coefficient = 0.40), and when combining parasites isolated from head‐thoraxes of louse flies in 2012 and 2013 (Jaccard coefficient = 0.37). A single parasite lineage, *Plasmodium *LK6, was shared between louse flies and Eleonora's falcons, although louse flies are not competent vectors of *Plasmodium*. Falcons and kills, however, did not share any parasite lineage (Table 1, Figure 1).

Sixteen of the parasite lineages isolated from the louse flies had previously been found infecting avian species (see Table S2 of the Supplemental Information). Bird kills had seven parasite lineages that were not found in louse flies, including the *Plasmodium* lineages COLL1, GRW9, and MOALB1, and the newly described *Haemoproteus* lineages HIPOL5, PHYBON1, PHYTRO1 and SYCAN02. In contrast, two of the new lineages described here (i.e., LANSEN1 and HIPOL4) were also shared with louse flies (Table 1, Figure 1).

From the 19 abdomens of louse flies with positive amplifications, DNA from birds of the *Falco *genus and *Hippolais polyglotta* was amplified from 11 and three louse flies, respectively*.* It was not possible to identify the blood meals of the remaining five louse flies, probably because of degradation of the host DNA.

**Figure 2:**
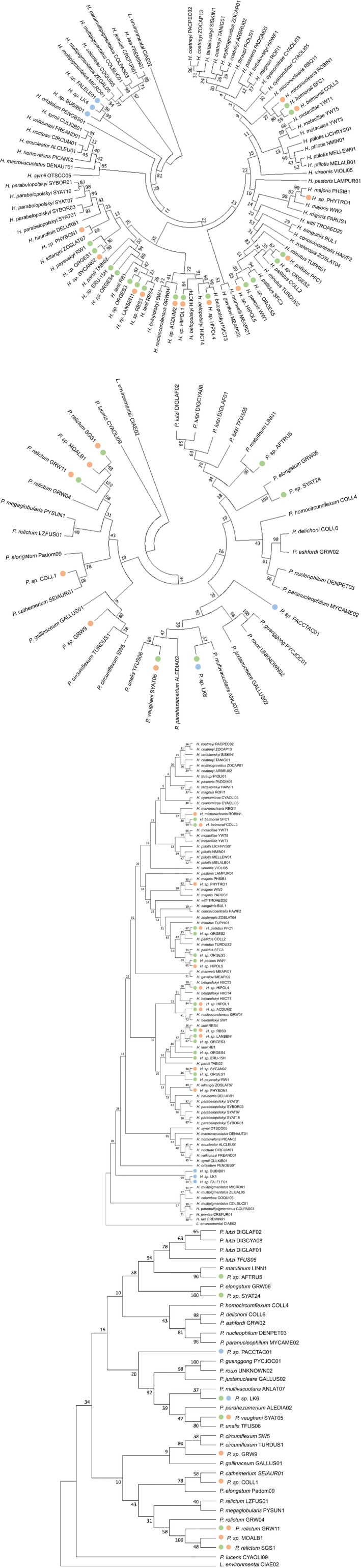
Bootstrap consensus tree inferred from 1,000 replications for the (a) *Plasmodium* and (b) *Haemoproteus* lineages found in falcon kills (orange), louse flies (green) and adult Eleonora's falcons (blue) with respect to available sequences from known morphospecies deposited in MalAvi (Bensch et al., 2009). The evolutionary history was inferred using the maximum likelihood method based on the Jukes‐Cantor model (Jukes & Cantor, 1969). Branches appearing in less than 50% of the bootstrap replicates were collapsed. Initial tree(s) for the heuristic search were automatically obtained by applying neighbour‐join and BioNJ algorithms to a matrix of pairwise distances estimated using the maximum composite likelihood approach, and then selecting the topology with best log‐likelihood value. The analysis included 112 nucleotide sequences. All positions containing gaps or missing data were eliminated, resulting in 461 positions in the final data set. Evolutionary analyses were conducted in MEGA7 (Kumar et al., 2016)

According to the phylogenetic analysis, the parasites found in louse flies in this study are closely related to those previously isolated from Passeriformes (Figure 2; see Table S2 of the Supplemental Information). The new lineages found in the louse flies were distributed in different clusters, including phylogenetically related lineages of the following known *Haemoproteus *morphospecies: ORGES2 and ORGES5 were closely related to lineage *pallidus*, while ORGES3, ORGES4, and LANSEN1 were closely related to *lanii*. The lineage ORGES1 was closely related to lineage SYCAN02, which was isolated from bird kills. The lineage HIPOL4, which was isolated from both louse flies and kills, was closely related to the morphospecies *belopolsky*. Of the lineages isolated only in bird kills, HIPOL5 was closely related to the morphospecies *palloris*, while the lineages PHYBON1 and PHYTRO1 clustered with the lineages of *killagoi* and *majoris*, respectively*.*

## 4 DISCUSSION

Because of the generally low prevalence and diversity of the hemosporidian parasites on islands (Hellgren, Križanauskienė, Hasselquist, & Bensch, 2011; Martínez‐de la Puente et al., 2017; Padilla, Illera, Gonzalez‐Quevedo, Villalba, & Richardson, 2017; Pérez‐Rodríguez, Ramírez, Richardson, & Pérez‐Tris, 2013; Sari, Klompen, & Parker, 2013) and the presence of only one potential vector species on the island, we expected to find an essentially simple host‐vector‐parasite system. However, we found that the opportunistic feeding behavior of louse flies resulted in an unexpected massive transmission of haemosporidians from kills to falcons’ louse flies. We found a high prevalence of parasites in both the passerine bird prey and the louse flies, and lineage sharing between them was moderate (see Figure 1). However, the lineages isolated in the louse flies and falcon kills did not match those previously found infecting adult falcons. Haemosporidian diversity of adult falcons was low and lineage sharing between falcons and louse flies was also extremely low, with only one shared lineage (*Plasmodium* LK6). However, louse flies are not competent vectors of *Plasmodium*, so Eleonora's falcons were probably infected with this parasite by mosquitoes outside the breeding grounds (see Gangoso et al., 2016). Haemosporidians need several days to develop in their vertebrate hosts and, consequently, the lack of infection in Eleonora's falcon nestlings could be due to their young age. However, high prevalence of haemoparasites has been found in nestlings even younger than 20 days old of many different bird species, including other Falconiformes (e.g., Calero‐Riestra & García, 2016; Hanel et al., 2016; Svobodová et al., 2015). This finding, together with the fact that lineage sharing between louse flies and adult falcons is virtually null, supports the idea that nestlings do not become infected at breeding grounds.

The success of transmission of a particular parasite lineage to a new host depends to a large extent upon the susceptibility of the host and compatibility between blood‐feeding insects and parasite lineages (Beerntsen, James, & Christensen, 2000; Martínez‐de la Puente, Martínez, Rivero‐de Aguilar, Herrero, & Merino, 2011). Indeed, only a fraction of the parasites that are in contact with potential vectors are effectively transmitted (Gutiérrez‐López et al., 2016), because this process may be hampered by environmental, behavioral, genetic, and physiological factors that inhibit the development of parasites in blood‐sucking insects (Beerntsen et al., 2000; Molina‐Cruz, Garver et al., 2013). For example, the immune system of the human malaria vector *Anopheles gambiae* is able to eliminate some strains of *Plasmodium falciparum*, while other strains can evade this immune barrier through the function of a particular parasite gene (Molina‐Cruz et al., 2012; Molina‐Cruz, Garver et al., 2013). However, some blood parasites may overcome the genetic and physiological barriers of new, often evolutionarily distant vectors, as has been shown for different avian *Plasmodium* spp. transmitted by anopheline and culicine mosquitoes under laboratory conditions (Molina‐Cruz, Lehmann, & Knöckel, 2013b; and references therein), and for *Plasmodium vivax* and different *Anopheles* species in their natural environments (Joy et al., 2008). Adapting to a new vector following environmental changes may be fuelled by the high evolutionary potential of blood parasites (Bensch, Pérez‐Tris, Waldenström, & Hellgren, 2004; Joy et al., 2008) and high mutations rates at some loci, such as those involved in the evasion of vector immune systems (Molina‐Cruz et al., 2015). In the parasite‐vector arms race, from the parasite's point of view, the benefits of adapting to a new, probably more abundant, vector must exceed the costs ensuing, as may be the reduced transmission efficiency in the original vector (Cohuet, Harris, Robert, & Fontenille, 2010). However, infection‐induced fitness costs should not reduce vector survival and prevent parasite transmission (Frank & Schmid‐Hempel, 2008). Although few studies have investigated this issue in the louse fly‐*Haemoproteus* system, Waite, Henry, Adler, and Clayton (2012) reported that the survival and fecundity of female *Pseudolynchia canariensis* flies decreased when feeding on birds infected with *H. columbae, *even though these flies effectively transmit the parasite. We do not know to what extent infections by blood parasites impose fitness costs to *O. gestroi*, or whether associated ecological and evolutionary processes affect susceptibility to the parasite and potential levels of transmission (Tripet, Aboagye‐Antwi, & Hurd, 2008; Wolinska & King, 2009). In any case, in a novel habitat, the continuous interaction between blood parasites and highly abundant potential vectors would increase the likelihood of vector switching.

**Figure 3:**
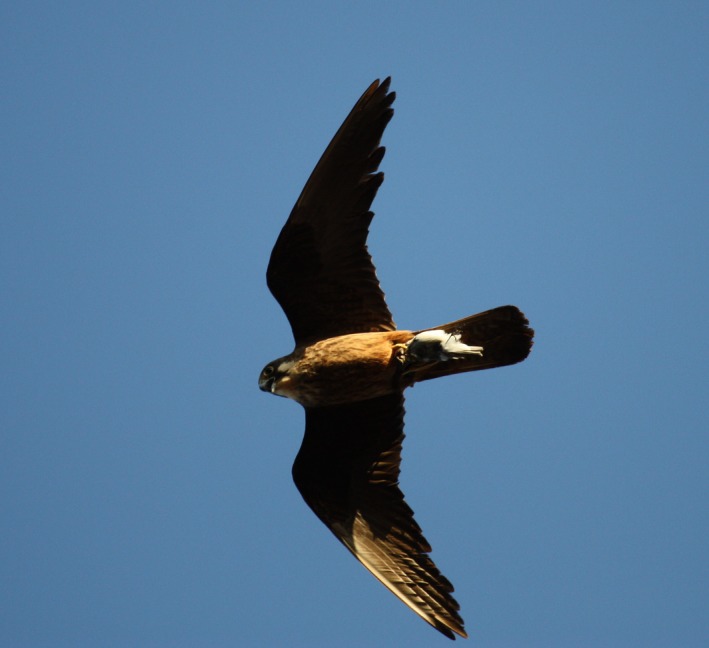
Male Eleonora's falcon carrying a passerine bird that is attached to the falcon's body during transport. Photo: Laura Gangoso

Contact rates between infected and new hosts are largely determined by insect feeding preferences and host specificity (Gager et al., 2008; Malmqvist, Strasevicius, Hellgren, Adler, & Bensch, 2004; Medeiros, Hamer, & Ricklefs, 2013; Whiteman, Matson, Bollmer, & Parker, 2006). Louse flies of the genus *Ornitophila* include only two species, *O. metallica* and *O. gestroi*, and while *O. metallica* has been recorded in different bird species and geographical regions (Maa, 1969), *O. gestroi* has a very limited host range. Despite the clear specificity of these obligate ectoparasites, we found molecular evidence that *O. gestroi* also feeds on passerines hunted by Eleonora's falcons, which may increase the contact rate with the blood parasites carried by these bird species and hence, the probability of parasite spillover. How do louse flies become infected? Migratory birds are hunted over the ocean by male Eleonora's falcons, and prey are subsequently transported to the breeding colony over distances of up to 50 km (Viana et al., 2016). During this journey, the prey is attached to the falcon's body (Figure 3), so louse flies have the opportunity to feed on the immobilized, usually still alive, bird. Subsequently, louse flies may be able to access nestlings from adult birds. Indeed, Levin and Parker (2014) reported that both infected and uninfected louse flies move between breeding adults within a frigatebird colony. It could be also possible that louse flies feed on fresh kills at the larders. However, we have never detected a louse fly feeding on a fresh prey or flying around larders, despite having handled thousands of falcon kills across years. In addition, kills are stored only when there is a surplus of food, a phenomenon that only occurs some days during the whole breeding season. Therefore, we believe that feeding on kills must occur while being transported. The high prevalence and diversity of lineages found in louse flies suggest that this opportunistic feeding behavior is rather common. In addition, louse flies may be involved in the transmission of *Haemoproteus* parasites between distantly related species, as in the case of seabirds (i.e., frigatebirds) and passerines (Jaramillo et al., 2017; Santiago‐Alarcon et al., 2014). The alternative hypothesis, that louse flies are carried by migratory passerines, is unlikely to be the case, as this louse fly species, to the best of our knowledge, has not been recorded on any other bird species than the above‐mentioned *Falco *species.

Despite the different sample sizes, we found that lineage sharing between louse flies and bird prey was moderately high. The most common parasites of louse flies, i.e., the *Haemoproteus *lineages PFC1 and HIPOL1, were found in the species that were the most frequently hunted by Eleonora's falcons, i.e., the European pied flycatcher and the common whitethroat. These findings, together with the fact that most of the parasite lineages isolated from louse flies corresponded to lineages infecting the Passeriformes (see Table S2 of the Supplemental Information), support the hypothesis that the origin of the parasites isolated from *O. gestroi* is bird prey hunted by falcons. The phylogenetic analysis revealed that the parasite lineages found in louse flies were related to lineages typical of the Passeriformes. Moreover, the new lineages isolated from louse flies in this study are closely related to lineages of *H. pallidus* and *H. lanii,* which are both common passerine parasites (according to MalAvi, Bensch et al., 2009), including of species that have been recorded as the prey of Eleonora's falcons (see Table 3 and Table S2 of the Supplemental Information). This suggests that louse flies are in continuous contact with a diverse array of parasite lineages by occasionally feeding on Eleonora's falcons’ bird prey. The *Haemoproteus* parasites that infect passerine birds throughout the world belong to the subgenus *Parahaemoproteus, *and are transmitted by *Culicoides* (Beadell et al., 2006; Martinsen, Perkins, & Schall, 2008). In contrast, louse flies transmit *Haemoproteus* parasites of the subgenus *Haemoproteus*, which infect Columbiformes and some seabird species (i.e., Suliformes and Charadriiformes), and a passerine (*Myiarchus magnirostris*) from the Galapagos archipelago (Levin et al., 2011; Sari et al., 2013; Valkiūnas et al., 2010). This suggests that life‐history traits of louse flies as compared to other insect vectors (e.g., obligate vs. free‐living ectoparasites) and ecological factors associated with insularity affect evolutionary relationships between geographically restricted hosts, potential insect vectors, and avian haemosporidians. It is possible that infections in louse flies do not contribute to parasite transmission beyond limiting parasite spread through the infection of a noncompetent vector. Finding parasites in the head‐thoraxes of louse flies suggests that the parasites are able to cross barriers and survive to some extent within louse flies. However, as we did not look for sporozoites, we cannot rule out the possibility that we amplified abortive infections in these louse flies (Valkiūnas, 2011; Valkiūnas et al., 2013), which would thus be dead‐end invertebrate hosts of these parasites or near‐successful, vector‐switching events.

It has been proposed that the main factor that inhibits the spread of haemosporidians across bird species is in the parasite‐host compatibility, as the vector carries a wide diversity of parasites but only a few succeed (Medeiros et al., 2013). A newly colonized host represents a novel habitat for the parasite, and probably has a new blood cellular and immunological profile that may hamper the ability of the parasite to invade host cells, or result in the abortive development of the parasite at the tissue stage (Olias et al., 2011; Valkiūnas et al., 2013). For example, Jaramillo et al. (2017) found that *H. multipigmentatus*, which is a parasite that is thought to be specific to columbiform birds (Valkiūnas et al., 2010), was able to infect six different species of passerines that co‐occurred with the parasite's main host, i.e., the endemic Galapagos dove. However, despite a successful spillover from introduced rock pigeons (*Columba livia*) to doves and the subsequent spillover from doves to passerines, the absence of parasite gametocytes in passerine bird blood suggests that these are not competent hosts for this *Haemoproteus* lineage (Jaramillo et al., 2017). Similarly, Moens et al. (2016) found gametocytes of the generalist *H. witti* only in Andean hummingbirds, and not in passerines that were probably infected by parasite spillover. The molecular detection of parasite stages, including sporozoites, in the birds’ peripheral blood could explain these results (Valkiūnas, Iezhova, Loiseau, & Sehgal, 2009).

Beyond the filtering effects exerted by parasite‐vector compatibility and parasite‐host compatibility, the host immune system could also prevent falcons from being infected by new parasite lineages. Alternatively, we cannot exclude a scenario of rare transmission events associated with high parasite virulence, with infected hosts being rapidly purged by high mortality (Poulin, 2006). The lack of parasites in Eleonora's falcon nestlings (Gutiérrez‐López, Gangoso et al., 2015a) and the mismatch between the lineages isolated here and those previously found in adult falcons (Gangoso et al., 2016) support the absence of successful transmission to this raptor species, despite louse flies being in contact with haemosporidians from a wide taxonomic range of avian species. This could be due to the falcons’ resistance to infection, the inability of parasites to develop in these phylogenetically distant species, and/or the inability of some parasite lineages to complete their development in louse flies.

## AUTHOR CONTRIBUTIONS

L.G., J.M.P., and J.F. designed the study. L.G. conducted the fieldwork and sampling. R.G.L. and J.M.P. performed the molecular determination of blood parasites. L.G., R.G.L., and J.M.P. analyzed the data. L.G. wrote the manuscript with input from all authors, who approved the final version.

## Data Availability

New sequences were deposited in the National Center for Biotechnology Information's GenBank database under the accession numbers MH271173‐MH271183.

## References

[mec15020-bib-0001] Agosta, S. J. , & Klemens, J. A. (2008). Ecological fitting by phenotypically flexible genotypes: Implications for species associations, community assembly and evolution. Ecology Letters, 11, 1123–1134. 10.1111/j.1461-0248.2008.01237.x 18778274

[mec15020-bib-0002] Alcaide, M. , Rico, C. , Ruiz, S. , Soriguer, R. , Muñoz, J. , & Figuerola, J. (2009). Disentangling vector‐borne transmission networks: A universal DNA barcoding method to identify vertebrate hosts from arthropod bloodmeals. PLoS ONE, 4, e7092 10.1371/journal.pone.0007092 19768113 PMC2740869

[mec15020-bib-0003] Alcala, N. , Jenkins, T. , Christe, P. , & Vuilleumier, S. (2017). Host shift and cospeciation rate estimation from co‐phylogenies. Ecology Letters, 20, 1014–1024. 10.1111/ele.12799 28662544

[mec15020-bib-0004] Bates, D. , Maechler, M. , Bolker, B. , & Walker, S. (2015). Fitting linear mixed‐effects models using lme4. Journal of Statistical Software, 67(1), 1812–48.

[mec15020-bib-0005] Beadell, J. S. , Ishtiaq, F. , Covas, R. , Melo, M. , Warren, B. H. , Atkinson, C. T. , … Rahmani, A. R. (2006). Global phylogeographic limits of Hawaii's avian malaria. Proceedings of the Royal Society of London B: Biological Sciences, 273, 2935–2944. 10.1098/rspb.2006.3671 PMC1639517 17015360

[mec15020-bib-0006] Beaucournu, J. C. , Beaucournu‐Saguez, F. , & Guiguen, C. (1985). Nouvelles données sur les diptères pupipares (Hippoboscidae et Streblidae) de la sous‐région mediterranéenne occidentale. Annales De Parasitologie Humaine Et Comparée, 60, 311–327.

[mec15020-bib-0007] Beerntsen, B. T. , James, A. A. , & Christensen, B. M. (2000). Genetics of mosquito vector competence. Microbiology and Molecular Biology Reviews, 64, 115–137. 10.1128/MMBR.64.1.115-137.2000 10704476 PMC98988

[mec15020-bib-0008] Bensch, S. , Hellgren, O. , & Pérez‐Tris, J. (2009). MalAvi: A public database of malaria parasites and related haemosporidians in avian hosts based on mitochondrial cytochrome b lineages. Molecular Ecology Resources, 9, 1353–1358. 10.1111/j.1755-0998.2009.02692.x 21564906

[mec15020-bib-0009] Bensch, S. , Pérez‐Tris, J. , Waldenström, J. , & Hellgren, O. (2004). Linkage between nuclear and mitochondrial DNA sequences in avian malaria parasites: Multiple cases of cryptic speciation? Evolution, 58, 1617–1621. 15341164 10.1111/j.0014-3820.2004.tb01742.x

[mec15020-bib-0010] Bobeva, A. , Zehtindjiev, P. , Bensch, S. , & Radrova, J. (2013). A survey of biting midges of the genus Culicoides Latreille, 1809 (Diptera: Ceratopogonidae) in NE Bulgaria, with respect to transmission of avian haemosporidians. Acta Parasitologica, 58, 585–591. 10.2478/s11686-013-0185-z 24338323

[mec15020-bib-0011] Calero‐Riestra, M. , & García, J. T. (2016). Sex‐dependent differences in avian malaria prevalence and consequences of infections on nestling growth and adult condition in the Tawny pipit, *Anthus campestris* . Malaria Journal, 15(1), 178 10.1186/s12936-016-1220-y 27001667 PMC4802721

[mec15020-bib-0012] Clark, N. J. , Clegg, S. M. , & Lima, M. R. (2014). A review of global diversity in avian haemosporidians (*Plasmodium* and *Haemoproteus*: Haemosporida): New insights from molecular data. International Journal for Parasitology, 44, 329–338. 10.1016/j.ijpara.2014.01.004 24556563

[mec15020-bib-0013] Cohuet, A. , Harris, C. , Robert, V. , & Fontenille, D. (2010). Evolutionary forces on Anopheles: What makes a malaria vector? Trends in Parasitology, 26, 130–136. 10.1016/j.pt.2009.12.001 20056485

[mec15020-bib-0014] R Core Team . (2017). R: A language and environment for statistical computing. Vienna, Austria: R Foundation for Statistical Computing Retrieved from https://www.R-project.org/

[mec15020-bib-0015] Drovetski, S. V. , Aghayan, S. A. , Mata, V. A. , Lopes, R. J. , Mode, N. A. , Harvey, J. A. , & Voelker, G. (2014). Does the niche breadth or trade‐off hypothesis explain the abundance–occupancy relationship in avian Haemosporidia? Molecular Ecology, 23, 3322–3329. 10.1111/mec.12744 24689968

[mec15020-bib-0016] Felsenstein, J. (1981). Evolutionary trees from DNA sequences: A maximum likelihood approach. Journal of Molecular Evolution, 17, 368–376. 10.1007/BF01734359 7288891

[mec15020-bib-0017] Ferraguti, M. , Martínez‐de la Puente, J. , Bensch, S. , Roiz, D. , Ruiz, S. , Viana, D. S. , … Figuerola, J. (2018). Ecological determinants of avian malaria infections: An integrative analysis at landscape, mosquito and vertebrate community levels. Journal of Animal Ecology, 87, 727–740. 10.1111/1365-2656.12805 29495129

[mec15020-bib-0018] Frank, S. A. , & Schmid‐Hempel, P. (2008). Mechanisms of pathogenesis and the evolution of parasite virulence. Journal of Evolutionary Biology, 21, 396–404. 10.1111/j.1420-9101.2007.01480.x 18179516

[mec15020-bib-0019] Gager, A. B. , Del Rosario Loaiza, J. , Dearborn, D. C. , & Bermingham, E. (2008). Do mosquitoes filter the access of Plasmodium cytochrome b lineages to an avian host? Molecular Ecology, 17, 2552–2561. 10.1111/j.1365-294X.2008.03764.x 18422926

[mec15020-bib-0020] Gangoso, L. , Grande, J. M. , Llorente, F. , Jiménez‐Clavero, M. A. , Pérez, J. M. , & Figuerola, J. (2010). Prevalence of neutralizing antibodies to West Nile virus in Eleonora's Falcons in the Canary Islands. Journal of Wildlife Diseases, 46, 1321–1324. 10.7589/0090-3558-46.4.1321 20966288

[mec15020-bib-0021] Gangoso, L. , Gutiérrez‐López, R. , Martínez‐de la Puente, J. , & Figuerola, J. (2016). Genetic colour polymorphism is associated with avian malarial infections. Biology Letters, 12, 20160839 10.1098/rsbl.2016.0839 28003524 PMC5206593

[mec15020-bib-0022] Gutiérrez‐López, R. , Gangoso, L. , Martínez‐de la Puente, J. , Fric, J. , López‐López, P. , Mailleux, M. , … Figuerola, J. (2015a). Low prevalence of blood parasites in a long‐distance migratory raptor: The importance of host habitat. Parasites & Vectors, 8, 189. 25889120 10.1186/s13071-015-0802-9 PMC4381668

[mec15020-bib-0023] Gutiérrez‐López, R. , Martínez‐de la Puente, J. , Gangoso, L. , Soriguer, R. C. , & Figuerola, J. (2015b). Comparison of manual and semi‐automatic DNA extraction protocols for the barcoding characterization of hematophagous louse flies (Diptera: Hippoboscidae). Journal of Vector Ecology, 40, 11–1825. 26047179 10.1111/jvec.12127

[mec15020-bib-0024] Gutiérrez‐López, R. , Martínez‐de la Puente, J. , Gangoso, L. , Yan, J. , Soriguer, R. C. , & Figuerola, J. (2016). Do mosquitoes transmit the avian malaria‐like parasite Haemoproteus? An experimental test of vector competence using mosquito saliva. Parasites & Vectors, 9, 609 10.1186/s13071-016-1903-9 27894354 PMC5127101

[mec15020-bib-0025] Hanel, J. , Doležalová, J. , Stehlíková, Š. , Modrý, D. , Chudoba, J. , Synek, P. , & Votýpka, J. (2016). Blood parasites in northern goshawk (*Accipiter gentilis*) with an emphasis to *Leucocytozoon toddi* . Parasitology Research, 115, 263–270. 10.1007/s00436-015-4743-1 26365666

[mec15020-bib-0026] Hellgren, O. , Križanauskienė, A. , Hasselquist, D. , & Bensch, S. (2011). Low haemosporidian diversity and one key‐host species in a bird malaria community on a mid‐Atlantic island (São Miguel, Azores). Journal of Wildlife Diseases, 47, 849–859. 10.7589/0090-3558-47.4.849 22102655

[mec15020-bib-0027] Hellgren, O. , Waldenström, J. , & Bensch, S. (2004). A new PCR assay for simultaneous studies of Leucocytozoon, Plasmodium, and Haemoproteus from avian blood. Journal of Parasitology, 90, 797–802. 10.1645/GE-184R1 15357072

[mec15020-bib-0028] Hiroshige, Y. , Hara, M. , Nagai, A. , Hikitsuchi, T. , Umeda, M. , Kawajiri, Y. , … Yamamoto, T. (2017). A human genotyping trial to estimate the post‐feeding time from mosquito blood meals. PLoS ONE, 12(6), e0179319 10.1371/journal.pone.0179319 28617865 PMC5472291

[mec15020-bib-0029] Ishtiaq, F. , Guillaumot, L. , Clegg, S. m. , Phillimore, A. b. , Black, R. a. , Owens, I. p. f. , … Sheldon, B. c. (2008). Avian haematozoan parasites and their associations with mosquitoes across Southwest Pacific Islands. Molecular Ecology, 17, 4545–4555. 10.1111/j.1365-294X.2008.03935.x 18986499

[mec15020-bib-0030] Jaccard, P. (1902). Lois de distribution florale dans la zone alpine. Bulletin De La Société Vaudoise Des Sciences Naturelles, 38, 69–130.

[mec15020-bib-0031] Jaramillo, M. , Rohrer, S. , & Parker, P. G. (2017). From Galapagos doves to passerines: Spillover of *Haemoproteus multipigmentatus* . International Journal for Parasitology: Parasites and Wildlife, 6, 155–161. 10.1016/j.ijppaw.2017.07.001 28736699 PMC5510524

[mec15020-bib-0032] Joy, D. A. , Gonzalez‐Ceron, L. , Carlton, J. M. , Gueye, A. , Fay, M. , McCutchan, T. F. , & Su, X. Z. (2008). Local adaptation and vector‐mediated population structure in *Plasmodium vivax* malaria. Molecular Biology and Evolution, 25, 1245–1252. 10.1093/molbev/msn073 18385220 PMC2386084

[mec15020-bib-0033] Jukes, T. H. , & Cantor, C. R. (1969). Evolution of protein molecules. Mammalian Protein Metabolism, 3, 132.

[mec15020-bib-0034] Kassara, C. , Gangoso, L. , Mellone, U. , Piasevoli, G. , Hadjikyriakou, T. G. , Tsiopelas, N. , … Gschweng, M. (2017). Current and future suitability of wintering grounds for a long‐distance migratory raptor. Scientific Reports, 7, 8798 10.1038/s41598-017-08753-w 28821735 PMC5562895

[mec15020-bib-0035] Kim, K. S. , & Tsuda, Y. (2010). Seasonal changes in the feeding pattern of *Culex pipiens pallens* govern the transmission dynamics of multiple lineages of avian malaria parasites in Japanese wild bird community. Molecular Ecology, 19, 5545–5554. 10.1111/j.1365-294X.2010.04897.x 21044196

[mec15020-bib-0036] Križanauskienė, A. , Hellgren, O. , Kosarev, V. , Sokolov, L. , Bensch, S. , & Valkiūnas, G. (2006). Variation in host specificity between species of avian hemosporidian parasites: Evidence from parasite morphology and cytochrome B gene sequences. Journal of Parasitology, 92, 1319–1324. 17304814 10.1645/GE-873R.1

[mec15020-bib-0037] Kumar, S. , Stecher, G. , & Tamura, K. (2016). MEGA7: Molecular evolutionary genetics analysis version 7.0 for bigger datasets. Molecular Biology and Evolution, 33, 1870–1874. 27004904 10.1093/molbev/msw054 PMC8210823

[mec15020-bib-0038] Lee, J. , Malmberg, J. L. , Wood, B. A. , Hladky, S. , Troyer, R. , Roelke, M. , … VandeWoude, S. (2017). Feline immunodeficiency virus cross‐species transmission: Implications for emergence of new lentiviral infections. Journal of Virology, 91, e02134–e2216. 10.1128/JVI.02134-16 28003486 PMC5309969

[mec15020-bib-0039] Levin, I. I. , & Parker, P. G. (2014). Infection with *Haemoproteus iwa* affects vector movement in a hippoboscid fly—frigatebird system. Molecular Ecology, 23, 947–953. 24215498 10.1111/mec.12587

[mec15020-bib-0040] Levin, I. I. , Valkiūnas, G. , Iezhova, T. A. , O'Brien, S. L. , & Parker, P. G. (2012). Novel Haemoproteus species (Haemosporida: Haemoproteidae) from the swallow‐tailed gull (Lariidae), with remarks on the host range of hippoboscid‐transmitted avian hemoproteids. Journal of Parasitology, 98, 847–854. 10.1645/GE-3007.1 22324933

[mec15020-bib-0041] Levin, I. I. , Valkiūnas, G. , Santiago‐Alarcon, D. , Cruz, L. L. , Iezhova, T. A. , O’Brien, S. L. , … Parker, P. G. (2011). Hippoboscid‐transmitted *Haemoproteus* parasites (Haemosporida) infect Galapagos Pelecaniform birds: Evidence from molecular and morphological studies, with a description of Haemoproteus iwa. International Journal for Parasitology, 41, 1019–1027. 10.1016/j.ijpara.2011.03.014 21683082

[mec15020-bib-0042] Maa, T. C. (1969). A revised checklist and concise host index of Hippoboscidae (Diptera). Pacific Insects Monograph, 20, 261–299.

[mec15020-bib-0043] Malmqvist, B. , Strasevicius, D. , Hellgren, O. , Adler, P. H. , & Bensch , S. (2004). Vertebrate host specificity of wild–caught blackflies revealed by mitochondrial DNA in blood. Proceedings of the Royal Society of London B: Biological Sciences, 271, S152–S155. 10.1098/rsbl.2003.0120 PMC1810009 15252969

[mec15020-bib-0044] Martínez‐de la Puente, J. , Eberhart‐Phillips, L. J. , Cristina Carmona‐Isunza, M. , Zefania, S. , Navarro, M. J. , Kruger, O. , … Figuerola, J. (2017). Extremely low *Plasmodium* prevalence in wild plovers and coursers from Cape Verde and Madagascar. Malaria Journal, 16, 243 10.1186/s12936-017-1892-y 28595600 PMC5465530

[mec15020-bib-0045] Martínez‐de la Puente, J. , Martínez, J. , Rivero‐de Aguilar, J. , Herrero, J. , & Merino, S. (2011). On the specificity of avian blood parasites: Revealing specific and generalist relationships between haemosporidians and biting midges. Molecular Ecology, 20, 3275–3287. 10.1111/j.1365-294X.2011.05136.x 21627703

[mec15020-bib-0046] Martínez‐de la Puente, J. , Muñoz, J. , Capelli, G. , Montarsi, F. , Soriguer, R. , Arnoldi, D. , … Figuerola, J. (2015). Avian malaria parasites in the last supper: Identifying encounters between parasites and the invasive Asian mosquito tiger and native mosquito species in Italy. Malaria Journal, 14, 32 10.1186/s12936-015-0571-0 25626918 PMC4318217

[mec15020-bib-0047] Martínez‐de la Puente, J. , Ruiz, S. , Soriguer, R. , & Figuerola, J. (2013). Effect of blood meal digestion and DNA extraction protocol on the success of blood meal source determination in the malaria vector *Anopheles atroparvus* . Malaria Journal, 12, 109 10.1186/1475-2875-12-109 23517864 PMC3608947

[mec15020-bib-0048] Martinsen, E. S. , Perkins, S. L. , & Schall, J. J. (2008). A three‐genome phylogeny of malaria parasites (*Plasmodium* and closely related genera): Evolution of life‐history traits and host switches. Molecular Phylogenetics and Evolution, 47, 261–273. 10.1016/j.ympev.2007.11.012 18248741

[mec15020-bib-0049] Medeiros, M. C. , Hamer, G. L. , & Ricklefs, R. E. (2013). Host compatibility rather than vector–host‐encounter rate determines the host range of avian Plasmodium parasites. Proceedings of the Royal Society of London B: Biological Sciences, 280, 20122947. 10.1098/rspb.2012.2947 PMC3652452 23595266

[mec15020-bib-0050] Medeiros, M. C. , Ellis, V. A. , & Ricklefs, R. E. (2014). Specialized avian Haemosporida trade reduced host breadth for increased prevalence. Journal of Evolutionary Biology, 27, 2520–2528. 10.1111/jeb.12514 25307516

[mec15020-bib-0051] Mendes, L. , Pardal, S. , Morais, J. , Antunes, S. , Ramos, J. A. , Perez‐Tris, J. , & Piersma, T. (2013). Hidden haemosporidian infections in Ruffs (*Philomachus pugnax*) staging in Northwest Europe en route from Africa to Arctic Europe. Parasitology Research, 112, 2037–2043. 10.1007/s00436-013-3362-y 23456021

[mec15020-bib-0052] Merino, S. , Hennicke, J. , Martínez, J. , Ludynia, K. , Torres, R. , Work, T. M. , … Quillfeldt, P. (2012). Infection by *Haemoproteus* parasites in four species of frigatebirds and the description of a new species of *Haemoproteus* (Haemosporida: Haemoproteidae). Journal of Parasitology, 98, 388–397. 10.1645/GE-2415.1 21992108

[mec15020-bib-0053] Moens, M. A. , Valkiūnas, G. , Paca, A. , Bonaccorso, E. , Aguirre, N. , & Pérez‐Tris, J. (2016). Parasite specialization in a unique habitat: Hummingbirds as reservoirs of generalist blood parasites of Andean birds. Journal of Animal Ecology, 85, 1234–1245. 10.1111/1365-2656.12550 27177277

[mec15020-bib-0054] Molina‐Cruz, A. , Canepa, G. E. , Kamath, N. , Pavlovic, N. V. , Mu, J. , Ramphul, U. N. , … Barillas‐Mury, C. (2015). Plasmodium evasion of mosquito immunity and global malaria transmission: The lock‐and‐key theory. Proceedings of the National Academy of Sciences, 112, 15178–15183. 10.1073/pnas.1520426112 PMC4679011 26598665

[mec15020-bib-0055] Molina‐Cruz, A. , DeJong, R. j. , Ortega, C. , Haile, A. , Abban, E. , Rodrigues, J. , … Barillas‐Mury, C. (2012). Some strains of Plasmodium falciparum, a human malaria parasite, evade the complement‐like system of Anopheles gambiae mosquitoes. Proceedings of the National Academy of Sciences, 109, E1957–E1962. 10.1073/pnas.1121183109 PMC3396512 22623529

[mec15020-bib-0056] Molina‐Cruz, A. , Garver, L. S. , Alabaster, A. , Bangiolo, L. , Haile, A. , Winikor, J. , … Barillas‐Mury, C. (2013). The human malaria parasite Pfs47 gene mediates evasion of the mosquito immune system. Science, 340, 984–987. 23661646 10.1126/science.1235264 PMC3807741

[mec15020-bib-0057] Molina‐Cruz, A. , Lehmann, T. , & Knöckel, J. (2013b). Could culicine mosquitoes transmit human malaria? Trends in Parasitology, 29, 530–537. 24140295 10.1016/j.pt.2013.09.003 PMC10987011

[mec15020-bib-0058] Olias, P. , Wegelin, M. , Zenker, W. , Freter, S. , Gruber, A. D. , & Klopfleisch, R. (2011). Avian malaria deaths in parrots, Europe. Emerging Infectious Diseases, 17, 950 10.3201/eid1705.101618 21529428 PMC3338161

[mec15020-bib-0059] Padilla, D. P. , Illera, J. C. , Gonzalez‐Quevedo, C. , Villalba, M. , & Richardson, D. S. (2017). Factors affecting the distribution of haemosporidian parasites within an oceanic island. International Journal for Parasitology, 47, 225–235. 10.1016/j.ijpara.2016.11.008 28161403

[mec15020-bib-0060] Palinauskas, V. , Valkiūnas, G. , Bolshakov, C. V. , & Bensch, S. (2008). *Plasmodium relictum* (lineage P‐SGS1): Effects on experimentally infected passerine birds. Experimental Parasitology, 120, 372–380. 10.1016/j.exppara.2008.09.001 18809402

[mec15020-bib-0061] Pérez‐Rodríguez, A. , Ramírez, A. , Richardson, D. S. , & Pérez‐Tris, J. (2013). Evolution of parasite island syndromes without long‐term host population isolation: Parasite dynamics in Macaronesian blackcaps *Sylvia atricapilla* . Global Ecology and Biogeography, 22, 1272–1281.

[mec15020-bib-0062] Poulin, R. (2006). Evolutionary ecology of parasites (2nd ed.). Princeton, NJ: Princeton University Press.

[mec15020-bib-0063] Real, R. (1999). Tables of significant values of Jaccard’s index of similarity. Miscel· Lania Zoologica, 22, 29–40.

[mec15020-bib-0064] Ricklefs, R. E. , Outlaw, D. C. , Svensson‐Coelho, M. , Medeiros, M. C. , Ellis, V. A. , & Latta, S. (2014). Species formation by host shifting in avian malaria parasites. Proceedings of the National Academy of Sciences, 111, 14816–14821. 10.1073/pnas.1416356111 PMC4205622 25271324

[mec15020-bib-0065] Santiago‐Alarcón, D. , Palinauskas, V. , & Schaefer, H. M. (2012). Diptera vectors of avian haemosporidian parasites: Untangling parasite life cycles and their taxonomy. Biological Reviews, 87, 928–964. 10.1111/j.1469-185X.2012.00234.x 22616880

[mec15020-bib-0066] Santiago‐Alarcon, D. , Rodríguez‐Ferraro, A. , Parker, P. G. , & Ricklefs, R. E. (2014). Different meal, same flavor: Cospeciation and host switching of haemosporidian parasites in some non‐passerine birds. Parasites & Vectors, 7, 286 10.1186/1756-3305-7-286 24957563 PMC4077843

[mec15020-bib-0067] Sari, E. H. , Klompen, H. , & Parker, P. G. (2013). Tracking the origins of lice, haemosporidian parasites and feather mites of the Galapagos flycatcher (*Myiarchus magnirostris*). Journal of Biogeography, 40, 1082–1093.

[mec15020-bib-0068] Sieber, M. , & Gudelj, I. (2014). Do‐or‐die life cycles and diverse post‐infection resistance mechanisms limit the evolution of parasite host ranges. Ecology Letters, 17, 491–498. 10.1111/ele.12249 24495077

[mec15020-bib-0069] Svobodová, M. , Weidinger, K. , Peške, L. , Volf, P. , Votýpka, J. , & Voříšek, P. (2015). Trypanosomes and haemosporidia in the buzzard (*Buteo buteo*) and sparrowhawk (*Accipiter nisus*): Factors affecting the prevalence of parasites. Parasitology Research, 114, 551–560. 10.1007/s00436-014-4217-x 25403377

[mec15020-bib-0070] Synek, P. , Munclinger, P. , Albrecht, T. , & Votýpka, J. (2013). Avian haemosporidians in haematophagous insects in the Czech Republic. Parasitology Research, 112, 839–845. 10.1007/s00436-012-3204-3 23224608

[mec15020-bib-0071] Takken, W. , & Verhulst, N. O. (2013). Host preferences of blood‐feeding mosquitoes. Annual Review of Entomology, 58, 433–453. 10.1146/annurev-ento-120811-153618 23020619

[mec15020-bib-0072] Tripet, F. , Aboagye‐Antwi, F. , & Hurd, H. (2008). Ecological immunology of mosquito – malaria interactions. Trends in Parasitology, 24, 219–227. 10.1016/j.pt.2008.02.008 18424235 PMC2474669

[mec15020-bib-0073] Valkiūnas, G. (2005). Avian malaria parasites and other haemosporidia. Boca Raton, FL: CRC Press.

[mec15020-bib-0074] Valkiūnas, G. (2011). Haemosporidian vector research: Marriage of molecular and microscopical approaches is essential. Molecular Ecology, 20, 3084–3086. 10.1111/j.1365-294X.2011.05187.x 21901870

[mec15020-bib-0075] Valkiūnas, G. , Iezhova, T. A. , Loiseau, C. , & Sehgal, R. N. (2009). Nested cytochrome b polymerase chain reaction diagnostics detect sporozoites of hemosporidian parasites in peripheral blood of naturally infected birds. Journal of Parasitology, 95, 1512–1515. 10.1645/GE-2105.1 19522549

[mec15020-bib-0076] Valkiūnas, G. , Kazlauskienė, R. , Bernotienė, R. , Palinauskas, V. , & Iezhova, T. A. (2013). Abortive long‐lasting sporogony of two *Haemoproteus* species (Haemosporida, Haemoproteidae) in the mosquito *Ochlerotatus cantans*, with perspectives on haemosporidian vector research. Parasitology Research, 112, 2159–2169. 10.1007/s00436-013-3375-6 23504040

[mec15020-bib-0077] Valkiūnas, G. , Santiago‐Alarcon, D. , Levin, I. I. , Iezhova, T. A. , & Parker, P. G. (2010). A new Haemoproteus species (Haemosporida: Haemoproteidae) from the endemic Galapagos dove *Zenaida galapagoensis*, with remarks on the parasite distribution, vectors, and molecular diagnostics. Journal of Parasitology, 96, 783–792. 10.1645/GE-2442.1 20486741

[mec15020-bib-0078] Viana, D. S. , Gangoso, L. , Bouten, W. , & Figuerola, J. (2016). Overseas seed dispersal by migratory birds. Proceedings of the Royal Society of London B: Biological Sciences, 283, 20152406. 10.1098/rspb.2015.2406 PMC4721096 26740610

[mec15020-bib-0079] Waite, J. L. , Henry, A. R. , Adler, F. R. , & Clayton, D. H. (2012). Sex‐specific effects of an avian malaria parasite on an insect vector: Support for the resource limitation hypothesis. Ecology, 93, 2448–2455. 10.1890/11-2229.1 23236915

[mec15020-bib-0080] Walter, H. (1979). Eleonora's falcon: Adaptations to prey and habitat in a social raptor. Chicago, IL, USA: University of Chicago Press.

[mec15020-bib-0081] Whiteman, N. K. , Matson, K. D. , Bollmer, J. L. , & Parker, P. G. (2006). Disease ecology in the Galapagos Hawk (Buteo galapagoensis): host genetic diversity, parasite load and natural antibodies. Proceedings of the Royal Society of London B: Biological Sciences, 273, 797–804. 10.1098/rspb.2005.3396 PMC1560217 16618672

[mec15020-bib-0082] Wolinska, J. , & King, K. C. (2009). Environment can alter selection in host–parasite interactions. Trends in Parasitology, 25, 236–244. 10.1016/j.pt.2009.02.004 19356982

[mec15020-bib-0083] Yan, J. , Gangoso, L. , Martínez‐de la Puente, J. , Soriguer, R. , & Figuerola, J. (2017). Avian phenotypic traits in relation to feeding preferences in two *Culex* mosquitoes. Science of Nature, 104, 76. 10.1007/s00114-017-1497-x 28856384

