# Supplementary material for: Louse flies of Eleonora’s falcons that also feed on their prey are evolutionary dead‐end hosts for blood parasites
Source: Mol Ecol. 2019 Apr 4;28(7):1812–25. doi: 10.1111/mec.15020 (PMC6850589; doi:10.1111/mec.15020)
Supplement: Supplementary file 2 [file MEC-28-1812-s002.pdf]

Electronic Supplementary Material

Louse flies of Eleonora's falcons that also feed on their prey are evolutionary dead-end hosts for blood parasites

Laura Gangoso, Rafael Gutiérrez-López, Josué Martínez-de la Puente & Jordi Figuerola

Table S2. *Haemoproteus* and *Plasmodium* parasite lineages found in this study. The different avian host where these lineages were isolated according to MalAvi public database (Bensch et al. 2009) are shown. The different continents are abbreviated as: "Af" = Africa, "Am" = America, "As" = Asia, "Eu" = Europe, and "Oce" = Oceania. The host species recorded as prey of Eleonora's falcons (*Falco eleonorae*) on the Canary Islands are highlighted with an *.

**Parasites**	**Bird hosts**						***F. eleonorae***
Parasite lineage	**Order**	**Family**	**Genus**	**Species**	**Continent**	**References**	**Prey**
Haemoproteus sp. ACDUM2	Passeriformes	Sylviidae	Acrocephalus	*A. agricola*	As, Eu	1, 2, 3	
				A. dumetorum	As	4	
			Hippolais	*H. polyglotta*	Eu	5, This study	*
			Sylvia	*S. communis*	Eu	This study	*
Haemoproteus sp. HIPOL4	Passeriformes	Sylviidae	Hippolais	*H. polyglotta*	Eu	This study	*
Haemoproteus sp. LANSEN1	Passeriformes	Laniidae	Lanius	*L. senator*	Eu	This study	*
		Muscicapidae	Ficedula	*F. hypoleuca*	Eu	This study	*
Haemoproteus sp. HIPOL5	Passeriformes	Phylloscopidae	Phylloscopus	*P. bonelli*	Eu	This study	*
		Sylviidae	Hippolais	*H. polyglotta*	Eu	This study	*
Haemoproteus attenuatus ROBIN1	Passeriformes	Laniidae	Lanius	*L. senator*	Eu	This study	*
		Turdidae	Erithacus	*E. rubecula*	Af, As, Eu	2, 4, 6, 7	*
			Luscinia	*L. luscinia*	As, Eu	4, 8, 9, 10	
				L. megarhynchos	Eu	2, 4, This study	*

			Luscinia	*L. luscinia*	As, Eu	4, 8, 9, 10	
				L. megarhynchos	Eu	2, 4, This study	*
			Saxicola	*S. rubetra*	Af, Eu	4	*
Haemoproteus balmorali COLL3	Passeriformes	Muscicapidae	Ficedula	*F. albicollis*	Eu	11	
				F. hypoleuca	As	12, This study	
				F. speculigera	Af	6	
			Muscicapa	*M. striata*	As	13	
Haemoproteus balmorali SFC1	Passeriformes	Muscicapidae	Muscicapa	*M. striata*	Af, As, Eu	2, 6, 13, 14, 15	*
		Paridae	Cyanistes	*C. caeruleus*	Eu	16, 17, 18, 19	
Haemoproteus sp. ERU-15H	Passeriformes	Laniidae	Lanius	*L. collurio*	Eu-As	Yindrim et al. unpubl	
Haemoproteus sp. HIPOL1	Passeriformes	Muscicapidae	Ficedula	*F. hypoleuca*	Eu	This study	*
		Sylviidae	Hippolais	*H. icterina*	Eu	5	
				H. polyglotta	Eu, Af	5, 6, This study	*
			Sylvia	*S. communis*	Eu	This study	*
		Ploceidae	Ploceus	*P. nigricollis*	Af	4	
Haemoproteus pallidus PFC1	Passeriformes	Fringillidae	Coccothraustes	*C. coccothraustes*	As	4	
		Muscicapidae	Ficedula	*F. albicollis*	Eu	11, 10, 21, 22	
				F. hypoleuca	Af, As, Eu	4, 12, 13, 14, 20, 24, 25, This study	*
		Phylloscopidae	Phylloscopus	*P. trochilus*	Eu	This study	*
		Sylviidae	Acrocephalus	*A. paludicola*	Eu	This study	*
			Sylvia	*S. cantillans*	Eu	This study	*
				S. communis	Eu	This study	*
Haemoproteus palloris WW1	Anseriformes	Anatidae	Cygnus	*C. olor*	Eu	26	
	Passeriformes	Muscicapidae	Ficedula	*F. hypoleuca*	As	12	
		Estrildidae	Uraeginthus	*U. bengalus*	Af	4	
		Panuridae	Panurus	*P. biarmicus*	As	Yildrim et al. unpubl	
		Paridae	Cyanistes	*C. caeruleus*	Eu	16, 17	
			Poecile	*P. montanus*	As	24	
		Ploceidae	Euplectes	*E. macroura*	Af	4	
		Phylloscopidae	Phylloscopus	*P. trochilus*	Af, As, Eu	4, 27, 28, 29, 30	*
		Sylviidae	Acrocephalus	*A. scirpaceus*	As	12	
			Sylvia	*S. borin*	Eu	31	*
	Upupiformes	Upupidae	Upupa	*U. epops*	As	32	
Haemoproteus payevsyi RW1	Passeriformes	Laniidae	Lanius	*L. meridionalis*	Eu	33	
		Cinclidae	Cinclus	*C. cinclus*	Eu	34	

		Cisticolidae	Cisticola	*C. nigriloris*	Af	29	
		Sylviidae	Acrocephalus	*A. baeticatus*	Af	29	
				A. scirpaceus	As, Eu	12, 13, 14, 23, 28, 35, 36	*
				A. schoenobaenus	Eu	36	
				A. palustris	As	4	
		Tudidae	Luscinia	*L. svecica*	Eu	37	*
Haemoproteus sp. RBS3	Passeriformes	Laniidae	Lanius	*L. collurio*	Eu	4	
				L. minor		2	
				L. senator	Eu	This study	*
Haemoproteus sp. ORGES1						This study	
Haemoproteus sp. ORGES2						This study	
Haemoproteus sp. ORGES3						This study	
Haemoproteus sp. ORGES4						This study	
Haemoproteus sp. ORGES5						This study	
Haemoproteus sp. PHYBON1	Passeriformes	Phylloscopidae	Phylloscopus	*P. bonelli*	Eu	This study	*
Haemoproteus sp. PHYTRO1	Passeriformes	Phylloscopidae	Phylloscopus	*P. trochilus*	Eu	This study	*
Haemoproteus sp. SYCAN02	Passeriformes	Sylviidae	Sylvia	*S. cantillans*	Eu	This study	*
Plasmodium AFTRU5	Passeriformes	Turdidae	Luscinia	*L. svecica*	As	38	
			Turdus	*T. merula*	Eu, Af	6, 39	
				T. pelios	Af	4, 40	
		Muscicapidae	Erithacus	*E. rubecula*	Eu	41	*
	Psitaciformes	Psittacidae	Melopsittacus	*M. undulatus*	Oce	42	
Plasmodium sp. LK6	Falconiformes	Falconidae	Falco	*F. eleonorae*	Eu	43, 44	
				F. naumanni	Eu	45, 46	
	Passeriformes	Alaudidae	Galerida	*G. cristata*	Af	6	
		Fringillidae	Emberiza	*E. cirlus*	Af	6	
			Serinus	*S. canarius*	Eu	41	
		Motacillidae	Anthus	*A. berthelotti*	Eu	41.47, 48	
				A. campestris	Eu	49	*
		Muscicapidae	Phoenicurus	*P. moussieri*	Af	6	
		Paridae	Periparus	*P. ater*	Af	6	
			Cyanistes	*C. teneriffae*	Af	6, 41	

		Phylloscopidae	Phylloscopus	*P. canariensis*	Eu	41	
		Sylviidae	Sylvia	*S. conspicillata*	Af, Eu	50	
				S. melanocephala	Af, Eu	6, 41	
Plamodium sp. MOALB1	Passeriformes	Alaudidae	Alauda	*A. arvensis*	Eu	51	
		Motacillidae	Motacilla	*M. alba*		4	*
				M. flava	Eu	This study	*
Plasmodium relictum GRW11	Charadriiformes	Scolopacidae	Philomachus	*P. pugnax*	Eu	52	
	Gruiformes	PhAsnidae	Lophophorus	*L. impejanus*	As	53	
			Chrysolophus	*C. amherstiae*	As	53	
	Passeriformes	Alaudidae	Alauda	*A. arvensis*	Eu	51	
		Certhiidae	Troglodytes	*T. troglodytes*	Eu	6, 54	
		Corvidae	Corvus	*C. corone*	As, Eu	55, 56	
				C. macrorhynchos	As	57	
			Garrulus	*G. glandarius*	As	54	
		Fringiliidae	Carduelis	*C. carduelis*	As	54	
			Emberiza	*E. cirlus*	Eu	6, 54	
			Fringilla	*F. coelebs*	As, Af, Eu	2, 6, 12, 54	
			Pyrrhula	*P. pyrrhula*	As	54	
		Muscicapidae	Ficedula	*F. albicollis*	Eu	11, 21, 25	
			Phoenicurus	*P. ochruros*	Eu	6, 54	*
		Hirundinidae	Delichon	*D. urbicum*	Af, Eu	58, 59, 60	
				D. dasypus	As	61	
		Laniidae	Lanius	*L. collurio*	Eu	2	
		Passeridae	Passer	*P. domesticus*	Af, Eu	2, 6, 28, 39, 54, 62, 63, 64, 65, 108	
				P. hispaniolensis	Af, Eu	2, 6, 54, 66	
				P. montanus	As, Eu	2, 66	
		Pariidae	Cyanistes	*C. caeruleus*	Eu	6, 16, 18, 19, 54, 67, 68, 69, 70	
				C. teneriffae	Af	6, 54	
			Parus	*P. major*	As, Eu	6, 7, 54, 71, 72	
			Periparus	*P. ater*	Af	6, 54	
		Pycnonotidae	Pycnonotus	*P. capensis*	Af	73	
		Sylviidae	Acrocephalus	*A. agricola*	Eu	1, 2	
				A.arundinaceus	Eu	2, 74	
				A. scirpaceus	Eu	28	*

			Cettia	*C. cetti*	Eu	28	
			Sylvia	*S. atricapilla*	Eu	4, 75	
				S. borin	As, Af, Eu	4, 12, 76	*
				S. cantillans	Eu	2	*
				S. communis	As	12, 54, This study	*
				S. conspicillata	Eu	50	
				S. curruca	As	12, 54	
				S. melanocephala	Eu	4	
				S. nisoria	As	54	
		Turdidae	Erithacus	*E. rubecula*	Eu	6, 54	*
			Luscinia	*L. luscinia*	Eu	4	
				L. svecica	Eu	37, 77	*
			Saxicola	*S. rubetra*	As	Yildrim et al. unpubl	
Plasmodium relictum SGS1 (Rinshi-1)	Anseriformes	Anatidae	Anas	*A. acuta*	Eu	78	
			Marmaronetta	*M. angustirostris*	Eu	78	
	Charadriiformes	Laridae	Larus	*L. argentatus*	Eu	79	
				L. cachinnans	Eu	79	
				L. mongolicus	As	80	
		Recurvirostridae	Himantopus	*H. himantopus*	Eu	81	
		Scolopacidae	Gallinago	*G. gallinago*	Eu	81	
	Ciconiiformes	Ardeidae	Botaurus	*B. stellaris*	As	82	
			Bubulcus	*B. ibis*	Eu	78	
		Ciconiidae	Ciconia	*C. ciconia*	Eu	78	
	Columbiformes	Columbidae	Columba	*C. livia*	Eu	83	
	Galliformes	PhAsnidae	Gallus	*G. gallus*	Eu	39	
			Lophophorus	*L. impejanus*	As	53	
			Perdix	*P. perdix*	Eu	84	
			Tragopan	*T. temminckii*	As	53	
	Gruiformes	Gruidae	Grus	*G. nigricollis*	As	85	
	Passeriformes	Alaudidae	Alauda	*A. arvensis*	Eu	51	
		Certhiidae	Certhia	*C. brachydactyla*	Af, Eu	6, 54	
			Troglodytes	*T. aedon*	S Am	86	
				T. troglodytes	Af, As, Eu	6, 54	
		Corvidae	Corvus	*C. corone*	As, Eu	55, 56, Scaglione et al. unpubl.	

				C. macrorhynchos	As	57	
			Cyanopica	*C. cooki*	Eu	6, 54	
			Garrulus	*G. glandarius*	As, Eu	2, 54, 87	
			Pica	*P. pica*	Eu	56	
		Estrildidae	Estrilda	*E. astrild*	Eu	28	
		Fringillidae	Carduelis	*C. chloris*	Af, As, Eu	6, 54, 87, 88	
				C. carduelis	As, Eu	2, 6, 54	
				C. spinus	As	89	
			Carpodacus	*C. erythrinus*	As, Eu	12, 54, 90	
			Coccothraustes	*C. coccothraustes*	As	91	
			Conirostrum	*C. cinereum*	S Am	86	
			Emberiza	*E. calandra*	As	54	
				E. cia	Eu	6, 54	
				E. cirlus	Eu	6, 54	
				E. citrinella	Oce	85, 92	
				E. elegans	As	93	
				E. godlewskii	As	93	
				E. hortulana	As	55	
				E. tahapisi	Af	4	
			Fringilla	*F. coelebs*	Af, As, Eu	6, 41, 54, 88	
			Loxia	*L. curvirostra*	As	89	
			Pyrrhula	*P. pyrrhula*	Eu	6, 54	
			Serinus	*S. canaria*	Eu	94	
				S. serinus	Af, Eu	6, 54	
			Zonotrichia	*Z. capensis*	S Am	86	
		Furnariidae	Phleocryptes	*P. melanops*	S Am	86	
		Hirundinidae	Delichon	*D. urbicum*	Af, Eu	58, 60	
		Laniidae	Lanius	*L. collaris*	Af	95	
				L. senator	Eu	This study	*
		Motacillidae	Motacilla	*M. flava*	Eu	4, 96	*
		Muscicapidae	Cercotrichas	*C. coryphoeus*	Af	95	
				C. galactotes	Af	4, 6, 54	*
				C. podobe	Af	4	
			Ficedula	*F. albicollis*	Eu	20, 21, 97	

				F. hypoleuca	As, Eu	12, 20, 24, 98	*
			Muscicapa	*M. striata*	Af, As	4, 6, 54	*
			Oenanthe	*O. oenanthe*	Af	54	*
			Phoenicurus	*P. moussieri*	Af	6, 54	
				P. ochruros	Eu	6, 54	*
				P. phoenicurus	As, Eu	2, 54	*
		Paridae	Cyanistes	*C. caeruleus*	As, Eu	6, 16, 17, 18, 19, 54, 67, 68, 69, 70, 72, 99, 100	
				C. teneriffae	Af	6, 54	
			Lophophanes	*L. cristatus*	Eu	6, 54	
			Parus	*P. major*	Af, As, Eu	6, 7, 12, 54, 57, 71, 72, 87, 93, 98, 101, 102, 103	
				P. venustulus	As	93	
				P. palustris	As	93	
			Periparus	*P. ater*	Af, Eu	6, 54, 98	
			Poecile	*P. montanus*	As	93	
				P. varius	As	87	
		Passeridae	Passer	*P. domesticus*	Af, As, Eu, Oce	2, 6, 28, 39, 54, 55, 62, 63, 64, 65, 92, 110	
				P. griseus	Af	Bensch and Ottosson unpubl	
				P. hispaniolensis	Eu	85	
				P. luteus	Af	85	
				P. melanurus	Af	95	
				P. montanus	As, Eu	2, 39, 57, 85	
				P. rufocinctus	Af	85	
			Prunella	*P. modularis*	As	54	
		Phylloscopidae	Phylloscopues	*P. bonelli*	Eu	This study	*
		Ploceidae	Euplectes	*E. orix*	Af	95	
			Ploceus	*P. capensis*	Af	95	
				P. melanocephalus	Eu	104	
				P. velatus	Af	95	
		Pycnonotidae	Microscelis	*M. amaurotis*	As	105	
			Pycnonotus	*P. capensis*	Af	73	
			Hypsipetes	*H. amaurotis*	As	53	
		Sittidae	Sitta	*S. europaea*	As	93	

		Sturnidae	Sturnus	*S. cineraceus*	As	105	
				S. tristis	As, Oce	87	
		Sylviidae	Acrocephalus	*A. agricola*	Eu	1, 2	
				A. arundinaceus	Eu	2, 28, 74, 106	
				A. palustris	As	4	
				A. schoenobaenus	Af	85	
				A. scirpaceus	Af, As, Eu	28, 85, 107	*
			Cettia	*C. cetti*	As, Eu	6, 28, 54	
			Hippolais	*H. polyglotta*	Eu	4, 6, 54	*
			Sylvia	*S. atricapilla*	Eu	4, 6, 41, 54, 75, 108	
				S. borin	Af, As, Eu	4, 6, 31, 54, 76, 89	*
				S. communis	As, Eu	4, 12, 54, This study	*
				S. curruca	Af, As	4, 54, 85	
				S. deserticola	Af	6, 54	
				S. melanocephala	Af, Eu	4, 6, 54	
				S. nisoria	Eu	2	
				S. undata	Eu	6, 54	
		Turdidae	Erithacus	*E. rubecula*	As, Eu	4, 6, 54	*
			Luscinia	*L. svecica*	Eu, As	8, 37, 77	*
			Monticola	*M. saxatilis*	As	54	
			Saxicola	*S. rubetra*	Af, Eu	4	*
				S. maura	As	54	
			Turdus	*T. merula*	Eu	39	
				T. viscivorus	Af	6, 54	
		Tyrannidae	Sayornis	*S. nigricans*	S Am	86	
			Serpophaga	*S. cinerea*	S Am	86	
	Procellariformes	Procellariidae	Pachyptila	*P. belcheri*	S Am	109	
	Spheniciformes	Spheniscidae	Spheniscus	S. humboldti	As	53	
	Strigiformes	Strigidae	Athene	*A. noctua*	Eu	6, 54	
	Trochiliformes	Trochilidae	Amazilia	*A. chionogaster*	S Am	86	
			Colibri	*C. coruscans*	S Am	86	
Plasmodium sp. COLL1	Passeriformes	Fringillidae	Emberiza	*E. cia*	Eu	6	
				E. cirlus	Eu	6	
		Motacillidae	Anthus	*A. campestris*	Eu	49	*
			Motacilla	*M. flava*	Eu	2, 4, 96	*

		Paridae	Cyanistes	*C. caeruleus*	Eu	19, 68	
		Passeridae	Passer	*P. domesticus*	Eu	2, 62, 64, 110	
				P. hispaniolensis	Eu	2, 66	
		Ploceidae	Euplectes	*E. orix*	Af	95	
		Sylviidae	Acrocephalus	*A. paludicola*	Eu	This study	*
			Locustella	*L. luscinioides*	Eu	28	
			Sylvia	*S. atricapilla*	Eu	4, 75	
Plasmodium sp. GRW9	Passeriformes	Cisticolidae	Camaroptera	*C. brachyura*	Af	4, 40, 87	
		Dicruridae	Dicrurus	*D. adsimilis*	Af	40, 87	
			Rhipidura	*R. rufifrons*	Oce	87	
		Estrildidae	Estrilda	*E. astrild*	Af	87	
				E. melpoda	Af	Loiseau unpubl	
			Lonchura	*L. cucullata*	Af	Loiseau unpubl	
			Pyrenestes	*P. ostrinus*	Af	40, 87	
			Spermophaga	*S. haematina*	Af	40, 87	
			Uraeginthus	*U. angolensis*	Af	29	
		Fringillidae	Crithagra	*C. capistrata*	Af	Loiseau unpubl	
			Serinus	*S. mozambicus*	Af	29	
		Hirundinidae	Delichon	*D. urbicum*	Eu	59, 60	
			Hirundo	*H. rustica*	Eu	2	
			Phedina	*P. borbonica*	Af	111	
			Psalidoprocne	*P. albiceps*	Af	29	
		Laniidae	Lanius	*L. collurio*	Eu	4	
		Motacillidae	Motacilla	*M. flaviventris*	Af	111	
		Muscicapidae	Copsychus	*C. albospecularis*	Afica	111	
			Cossypha	*C. heuglini*	Af	29	
				C. niveicapilla	Af	40, 87	
			Ficedula	*F. albicollis*	Eu	11, 20, 21, 22, 25, 97	
				F. hypoleuca	As, Eu	12, 20, 98, This study	*
				F. semitorquata	Eu	112	
			Muscicapa	*M. olivascens*	Af	40, 87	
			Pogonocichla	*P. stellata*	Af	29	
		Nectariniidae	Chalcomitra	*C. rubescens*	Af	40, 87	
			Cinnyris	*C. chloropygius*	Af	113	
				C. fuelleborni	Af	73	

				C. mediocris	Af	113	
			Cyanomitra	*C. olivacea*	Af	40, 73, 87, 113, 114	
				C. verticalis	Af	40, 87	
			Deleomis	*D. fraseri*	Af	40, 87	
			Hedydipna	*H. collaris*	Af	40, 87, 113	
			Nectarinia	*N. chloropugia*	Af	73	
		Passeridae	Gymnoris	*G. supercilliaris*	Af	29	
		Philepittidae	Philepitta	*P. castanea*	Af	111	
		Platysteiridae	Batis	*B. mixta*	Af	73	
		Ploceidae	Euplectes	*E. hordeaceus*	Af	Loiseau unpubl	
			Foudia	*F. hypoleuca*	Af	111	
				F. madagascariensis	Af	111	
			Malimbus	*M. nitens*	Af	40, 87	
			Ploceus	*P. cucullatus*	Af	40, 87	
				P. nelicourvi	Af	111	
			Quelea	*Q. quelea*	Af	29	
		Pycnonotidae	Andropadus	*A. chlorigula*	Af	73	
				A. gracilis	Af	40, 87	
				A. latirostris	Af	115, 116	
				A. masukuensis	Af	73	
				A. milanjensis	Af	29, 73	
				A. virens	Af	40, 87	
			Bernieria	*B. madagascariensis*	Af	111	
			Bleda	*B. eximius*	Af	4, 40, 87	
				B. notatus	Af	87	
				B. syndactylus	Af	40, 87	
			Criniger	*C. calurus*	Af	40, 87	
				C. chloronotus	Af	40, 87	
			Hypsipetes	*H. madagascariensis*	Af	111	
			Nicator	*N. chloris*	Af	40, 87	
			Phyllastrephus	*P. icterinus*	Af	40	
			Pycnonotus	*P. barbatus*	Af	4	

		Sylviidae	Acrocephalus	*A. arundinaceus*	Eu	106	
			Bradypterus	*B. baboecala*	Af	29	
			Nesillas	*N. typica*	Af	111	
			Sylvia	*S. borin*	Eu	4	*
		Timallidae	Modulatrix	*M. stictigula*	Af	73	
		Turdidae	Alethe	*A. fuelleborni*	Af	73	
				A. poliocephala	Af	40, 87	
			Neocossyphus	*N. fraseri*	Af	40, 87	
				N. poensis	Af	40, 87	
				N. rufus	Af	40, 87	
			Saxicola	*S. rubetra*	Eu	4	*
				S. torquata	Af	111	
			Sheppardia	*S. sharpei*	Af	73	
			Stiphrornis	*S. erythrothorax*	Af	40, 87	
			Zoothera	*Z. camaronensis*	Af	40, 87	
Plasmodium sp. SYAT24	Passeriformes	Sylviidae	Sylvia	*S. atricapilla*	Eu	4, 75	
Plasmodium vaughani SYAT05 (Rinshi-11)	Columbiformes	Columbidae	Hemiphaga	*H. novaeseelandiae*	Oce	117	
	Passeriformes	Alaudidae	Alauda	*A. arvensis*	Eu	51	
		Cinclidae	Cinclus	*C. cinclus*	Eu	34	
		Fringillidae	Fringilla	*F. coelebs*	Eu	118	
		Melipgagidae	Anthornis	*A. melanura*	Oce	119	
		Muscicapidae	Ficedula	*F. parva*	Eu	2	
		Paridae	Cyanistes	*C. caeruleus*	Eu	6, 54	
			Parus	*P. major*	Eu	7	
		Petroicidae	Petroica	*P. australis*	Oce	120	
				P. macrocephala	Oce	92	
		Sturnidae	Sturnus	*S. unicolor*	Eu	6, 54	
		Sylviidae	Cettia	*C. cetti*	Eu	28	
			Sylvia	*S. atricapilla*	Eu	6, 54, 108, 121	
				S. borin	Eu	31	*
				S. communis	Eu	This study	*
				S. malanocephala	Eu	4	
		Turdidae	Erythacus	*E. rubecula*	Eu	118	*
			Saxicola	*S. maura*	As	54	

			Turdus	*T. merula*	Af, As, Eu, Oce	2, 6, 7, 41, 54, 92, 117, 118, 120	
				T. migratorius	N Am	38, 122	
				T. pelios	Af	Loiseau unpubl	
				T. philomelos	Eu	6, 7, 54	
				T. viscivorus	Af	6, 54	
		Zosteropidae	Zosterops	*Z. lateralis*	Oce	120	
	Psittaciformes	Psittacidae	Melopsittacus	*M. undulatus*	Oce	42	

1. Zehtindjiev, P., Ilieva, M., Križanauskienė, A., Oparina, O., Oparin, M., & Bensch, S. (2009). Occurrence of haemosporidian parasites in the paddyfield warbler, *Acrocephalus agricola* (Passeriformes, Sylviidae). *Acta Parasitologica*, *54*(4), 295-300.
2. Dimitrov, D., Zehtindjiev, P., & Bensch, S. (2010). Genetic diversity of avian blood parasites in SE Europe: Cytochrome b lineages of the genera *Plasmodium* and *Haemoproteus* (Haemosporida) from Bulgaria. *Acta Parasitologica*, *55*(3), 201-209.
3. Ishtiaq, F. (2017). Exploring host and geographical shifts in transmission of haemosporidians in a Palaearctic passerine wintering in India. *Journal of Ornithology*, *158*(3), 869-874.
4. Hellgren, O., Waldenström, J., Pérez-Tris, J., Szöll, E., Si, Ö., Hasselquist, D., Krizanauskiene, A., Ottosson, U. & Bensch, S. (2007). Detecting shifts of transmission areas in avian blood parasites—a phylogenetic approach. *Molecular Ecology*, *16*(6), 1281-1290.
5. Reullier, J., Pérez-Tris, J., Bensch, S., & Secondi, J. (2006). Diversity, distribution and exchange of blood parasites meeting at an avian moving contact zone. *Molecular Ecology*, *15*(3), 753-763.
6. Mata, V. A., da Silva, L. P., Lopes, R. J., & Drovetski, S. V. (2015). The Strait of Gibraltar poses an effective barrier to host-specialised but not to host-generalised lineages of avian Haemosporidia. *International Journal for Parasitology*, *45*(11), 711-719.
7. Santiago-Alarcon, D., MacGregor-Fors, I., Kühnert, K., Segelbacher, G., & Schaefer, H. M. (2016). Avian haemosporidian parasites in an urban forest and their relationship to bird size and abundance. *Urban Ecosystems*, *19*(1), 331-346.
8. Hellgren, O. (2005). The occurrence of haemosporidian parasites in the Fennoscandian bluethroat (*Luscinia svecica*) population. *Journal of Ornithology*, *146*(1), 55-60.
9. Valkiūnas, G., Kazlauskienė, R., Bernotienė, R., Palinauskas, V., & Iezhova, T. A. (2013). Abortive long-lasting sporogony of two *Haemoproteus* species (Haemosporida, Haemoproteidae) in the mosquito *Ochlerotatus cantans*, with perspectives on haemosporidian vector research. *Parasitology Research*, *112*(6), 2159-2169.

10. Nilsson, E., Taubert, H., Hellgren, O., Huang, X., Palinauskas, V., Markovets, M.Y., Valkiūnas, G. & Bensch, S. (2016). Multiple cryptic species of sympatric generalists within the avian blood parasite *Haemoproteus majoris*. *Journal of Evolutionary Biology*, *29*(9), pp.1812-1826.
11. Radwan, J., Zagalska-Neubauer, M., Cichoń, M., Sendecka, J., Kulma, K., Gustafsson, L., & Babik, W. (2012). MHC diversity, malaria and lifetime reproductive success in collared flycatchers. *Molecular Ecology*, *21*(10), 2469-2479.
12. Križanauskienė, A., Hellgren, O., Kosarev, V., Sokolov, L., Bensch, S., & Valkiūnas, G. (2006). Variation in host specificity between species of avian hemosporidian parasites: evidence from parasite morphology and cytochrome b gene sequences. *Journal of Parasitology*, *92*(6), 1319-1324.
13. Hellgren, O., Križanauskiene, A., Valkiūnas, G., & Bensch, S. (2007). Diversity and phylogeny of mitochondrial cytochrome B lineages from six morphospecies of avian *Haemoproteus* (Haemosporida: Haemoproteidae). *Journal of Parasitology*, *93*(4), 889-896.
14. Valkiūnas, G., Palinauskas, V., Križanauskienė, A., Bernotienė, R., Kazlauskienė, R., & Iezhova, T. A. (2013). Further observations on in vitro hybridization of hemosporidian parasites: patterns of ookinete development in *Haemoproteus* spp. *Journal of Parasitology*, *99*(1), 124-136.
15. Valkiūnas, G., Iezhova, T. A., Križanauskienė, A., Palinauskas, V., & Bensch, S. (2008). In vitro hybridization of *Haemoproteus* spp.: an experimental approach for direct investigation of reproductive isolation of parasites. *Journal of Parasitology*, *94*(6), 1385-1394.
16. Wood, M. J., Cosgrove, C. L., Wilkin, T. A., Knowles, S. C., Day, K. P., & Sheldon, B. C. (2007). Within‐population variation in prevalence and lineage distribution of avian malaria in blue tits, *Cyanistes caeruleus*. *Molecular Ecology*, *16*(15), 3263-3273.
17. Cosgrove, C. L., Wood, M. J., Day, K. P., & Sheldon, B. C. (2008). Seasonal variation in Plasmodium prevalence in a population of blue tits *Cyanistes caeruleus*. *Journal of Animal Ecology*, *77*(3), 540-548.
18. Knowles, S. C., Wood, M. J., Alves, R., Wilkin, T. A., Bensch, S., & Sheldon, B. C. (2011). Molecular epidemiology of malaria prevalence and parasitaemia in a wild bird population. *Molecular Ecology*, *20*(5), 1062-1076.

19. Szöllősi, E., Cichoń, M., Eens, M., Hasselquist, D., Kempenaers, B., Merino, S., Nilsson, J.Å., Rosivall, B., Rytkönen, S., Török, J. & Wood, M.J. (2011). Determinants of distribution and prevalence of avian malaria in blue tit populations across Europe: separating host and parasite effects. *Journal of Evolutionary Biology*, *24*(9), pp.2014-2024.
20. Kulma, K., Low, M., Bensch, S., & Qvarnström, A. (2013). Malaria infections reinforce competitive asymmetry between two Ficedula flycatchers in a recent contact zone. *Molecular Ecology*, *22*(17), 4591-4601.
21. Kulma, K., Low, M., Bensch, S., & Qvarnström, A. (2014). Malaria-infected female collared flycatchers (*Ficedula albicollis*) do not pay the cost of late breeding. *PloS One*, *9*(1), e85822.
22. Garamszegi, L.Z., Zagalska-Neubauer, M., Canal, D., Markó, G., Szász, E., Zsebők, S., Szöllősi, E., Herczeg, G. & Török, J. (2015). Malaria parasites, immune challenge, MHC variability, and predator avoidance in a passerine bird. *Behavioral Ecology*, *26*(5), 1292-1302.
23. Križanauskienė, A., Iezhova, T. A., Palinauskas, V., Chernetsov, N., & Valkiūnas, G. (2012). Haemoproteus nucleocondensus n. sp.(Haemosporida, Haemoproteidae) from a Eurasian songbird, the Great Reed Warbler *Acrocephalus arundinaceus*. *Zootaxa*, *3441*(1), 36-46.
24. Palinauskas, V., Iezhova, T.A., Križanauskienė, A., Markovets, M.Y., Bensch, S. & Valkiūnas, G. (2013). Molecular characterization and distribution of *Haemoproteus minutus* (Haemosporida, Haemoproteidae): a pathogenic avian parasite. *Parasitology International,* 62(4), 358-363.
25. Szöllősi, E., Garamszegi, L. Z., Hegyi, G., Laczi, M., Rosivall, B., & Török, J. (2016). Haemoproteus infection status of collared flycatcher males changes within a breeding season. *Parasitology Research*, *115*(12), 4663-4672.
26. Wood, M. J., Childs, D. Z., Davies, A. S., Hellgren, O., Cornwallis, C. K., Perrins, C. M., & Sheldon, B. C. (2013). The epidemiology underlying age‐related avian malaria infection in a long‐lived host: the mute swan *Cygnus olor*. *Journal of Avian Biology*, *44*(4), 347-358.
27. Bensch, S., & Åkesson, S. (2003). Temporal and spatial variation of hematozoans in Scandinavian willow warblers. *Journal of Parasitology*, *89*(2), 388-391.

28. Ventim, R., Morais, J., Pardal, S., Mendes, L., Ramos, J. A., & Perez-Tris, J. (2012). Host-parasite associations and host-specificity in haemoparasites of reed bed passerines. *Parasitology*, *139*(3), 310-316.
29. Lutz, H.L., Hochachka, W.M., Engel, J.I., Bell, J.A., Tkach, V.V., Bates, J.M., Hackett, S.J. & Weckstein, J.D. (2015). Parasite prevalence corresponds to host life history in a diverse assemblage of afrotropical birds and haemosporidian parasites. *PloS One*, *10*(4), p.e0121254.
30. Dimitrov, D., Iezhova, T.A., Zehtindjiev, P., Bobeva, A., Ilieva, M., Kirilova, M., Bedev, K., Sjöholm, C. & Valkiūnas, G. (2016). Molecular characterisation of three avian haemoproteids (Haemosporida, Haemoproteidae), with the description of *Haemoproteus* (Parahaemoproteus) *palloris* n. sp. *Systematic Parasitology*, *93*(5), 431-449.
31. López, G., Muñoz, J., Soriguer, R., & Figuerola, J. (2013). Increased endoparasite infection in late-arriving individuals of a trans-Saharan passerine migrant bird. *PloS One*, *8*(4), e61236.
32. Zehtindjiev, P., Ivanova, K., Mariaux, J., & Georgiev, B. B. (2013). First data on the genetic diversity of avian haemosporidians in China: cytochrome b lineages of the genera *Plasmodium* and *Haemoproteus* (Haemosporida) from Gansu Province. *Parasitology Research*, *112*(10), 3509-3515.
33. Casanueva, P., Fernández, M., Ángeles Rojo, M., & Campos, F. (2012). High prevalence of haemosporidian parasites infection in southern grey shrike *Lanius meridionalis* (Laniidae, Aves) from agricultural areas. *Italian Journal of Zoology*, *79*(2), 315-318.
34. Rojo, M. Á., Hernández, M. Á., Campos, F., Santamaría, T., Dias, S., & Casanueva, P. (2015). The Iberian Peninsula is an area of infection by *Haemoproteus payevskyi* and *Haemoproteus nucleocondensus* for the White-throated Dipper *Cinclus cinclus*. *Ardeola*, *62*(2), 373-382.
35. Bensch, S., Stjernman, M., Hasselquist, D., Örjan, Ö., Hannson, B., Westerdahl, H., & Pinheiro, R. T. (2000). Host specificity in avian blood parasites: a study of *Plasmodium* and *Haemoproteus* mitochondrial DNA amplified from birds. *Proceedings of the Royal Society of London B: Biological Sciences*, *267*(1452), 1583-1589.
36. Fernández, M., Rojo, M. Á., Casanueva, P., Carrión, S., Hernández, M. Á., & Campos, F. (2010). High prevalence of haemosporidians in Reed Warbler

Acrocephalus scirpaceus and Sedge Warbler *Acrocephalus schoenobaenus* in Spain. *Journal of Ornithology*, *151*(1), 27.

37. Rojo, M. Á., Campos, F., Santamaría, T., & Hernández, M. Á. (2014). Haemosporidians in Iberian bluethroats *Luscinia svecica*. *Ardeola*, *61*(1), 135-143.
38. Martinsen, E. S., Perkins, S. L., & Schall, J. J. (2008). A three-genome phylogeny of malaria parasites (*Plasmodium* and closely related genera): evolution of life-history traits and host switches. *Molecular Phylogenetics and Evolution*, *47*(1), 261-273.
39. Martínez-de la Puente, J., Muñoz, J., Capelli, G., Montarsi, F., Soriguer, R., Arnoldi, D., Rizzoli, A. & Figuerola, J. (2015). Avian malaria parasites in the last supper: identifying encounters between parasites and the invasive Asian mosquito tiger and native mosquito species in Italy. *Malaria Journal*, *14*(1), 32.
40. Beadell, J.S., Covas, R., Gebhard, C., Ishtiaq, F., Melo, M., Schmidt, B.K., Perkins, S.L., Graves, G.R. & Fleischer, R.C. (2009). Host associations and evolutionary relationships of avian blood parasites from West Africa. *International Journal for Parasitology*, *39*(2), 257-266.
41. Padilla, D. P., Illera, J. C., Gonzalez-Quevedo, C., Villalba, M., & Richardson, D. S. (2017). Factors affecting the distribution of haemosporidian parasites within an oceanic island. *International Journal for Parasitology*, *47*(4), 225-235.
42. Baron, H. R., Howe, L., Varsani, A., & Doneley, R. J. (2013). Disease screening of three breeding populations of adult exhibition budgerigars (*Melopsittacus undulatus*) in New Zealand reveals a high prevalence of a novel polyomavirus and avian malaria infection. *Avian Diseases*, *58*(1), 111-117.
43. Gutiérrez-López, R., Gangoso, L., Martínez-de la Puente, J., Fric, J., López-López, P., Mailleux, M., Muñoz, J., Touati, L., Samraoui, B. & Figuerola, J. (2015). Low prevalence of blood parasites in a long-distance migratory raptor: the importance of host habitat. *Parasites & Vectors*, *8*(1), 189.
44. Gangoso, L., Gutiérrez-López, R., Martínez-de la Puente, J., & Figuerola, J. (2016). Genetic colour polymorphism is associated with avian malarial infections. *Biology Letters*, *12*(12), 20160839.
45. Ortego, J., Calabuig, G., Cordero, P. J., & Aparicio, J. M. (2007). Genetic characterization of avian malaria (Protozoa) in the endangered lesser kestrel, *Falco naumanni*. *Parasitology Research*, *101*(4), 1153-1156.

46. Ortego, J., Cordero, P. J., Aparicio, J. M., & Calabuig, G. (2008). Consequences of chronic infections with three different avian malaria lineages on reproductive performance of Lesser Kestrels (*Falco naumanni*). *Journal of Ornithology*, *149*(3), 337-343.
47. Illera, J. C., Emerson, B. C., & Richardson, D. S. (2008). Genetic characterization, distribution and prevalence of avian pox and avian malaria in the Berthelot's pipit (*Anthus berthelotii*) in Macaronesia. *Parasitology Research*, *103*(6), 1435-1443.
48. Spurgin, L. G., Illera, J. C., Padilla, D. P., & Richardson, D. S. (2012). Biogeographical patterns and co-occurrence of pathogenic infection across island populations of Berthelot's pipit (*Anthus berthelotii*). *Oecologia*, *168*(3), 691-701.
49. Calero-Riestra, M., & García, J. T. (2016). Sex-dependent differences in avian malaria prevalence and consequences of infections on nestling growth and adult condition in the Tawny pipit, *Anthus campestris*. *Malaria Journal*, *15*(1), 178.
50. Illera, J. C., Fernández‐Álvarez, Á., Hernández‐Flores, C. N., & Foronda, P. (2015). Unforeseen biogeographical patterns in a multiple parasite system in Macaronesia. *Journal of Biogeography*, *42*(10), 1858-1870.
51. Zehtindjiev, P., Križanauskienė, A., Scebba, S., Dimitrov, D., Valkiūnas, G., Hegemann, A., Tieleman, B.I. & Bensch, S. (2012). Haemosporidian infections in skylarks (*Alauda arvensis*): a comparative PCR-based and microscopy study on the parasite diversity and prevalence in southern Italy and the Netherlands. *European Journal of Wildlife Research*, *58*(1), 335-344.
52. Mendes, L., Pardal, S., Morais, J., Antunes, S., Ramos, J. A., Perez-Tris, J., & Piersma, T. (2013). Hidden haemosporidian infections in Ruffs (*Philomachus pugnax*) staging in Northwest Europe en route from Africa to Arctic Europe. *Parasitology Research*, *112*(5), 2037-2043.
53. Ejiri, H., Sato, Y., Sawai, R., Sasaki, E., Matsumoto, R., Ueda, M., Higa, Y., Tsuda, Y., Omori, S., Murata, K. & Yukawa, M. (2009). Prevalence of avian malaria parasite in mosquitoes collected at a zoological garden in Japan. *Parasitology Research*, *105*(3), 629.
54. Drovetski, S. V., Aghayan, S. A., Mata, V. A., Lopes, R. J., Mode, N. A., Harvey, J. A., & Voelker, G. (2014). Does the niche breadth or trade‐off hypothesis

explain the abundance–occupancy relationship in avian Haemosporidia?. *Molecular Ecology*, *23*(13), 3322-3329.

55. Martinsen, E. S., Paperna, I., & Schall, J. J. (2006). Morphological versus molecular identification of avian Haemosporidia: an exploration of three species concepts. *Parasitology*, *133*(3), 279-288.
56. Schmid, S., Fachet, K., Dinkel, A., Mackenstedt, U., & Woog, F. (2017). Carrion crows (*Corvus corone*) of southwest Germany: important hosts for haemosporidian parasites. *Malaria Journal*, *16*(1), 369.
57. Kim, K. S., & Tsuda, Y. (2010). Seasonal changes in the feeding pattern of *Culex pipiens pallens* govern the transmission dynamics of multiple lineages of avian malaria parasites in Japanese wild bird community. *Molecular Ecology*, *19*(24), 5545-5554.
58. Marzal, A., Bensch, S., Reviriego, M., Balbontin, J., & De Lope, F. (2008). Effects of malaria double infection in birds: one plus one is not two. *Journal of Evolutionary Biology*, *21*(4), 979-987.
59. Piersma, T., & van der Velde, M. (2012). Dutch House Martins *Delichon urbicum* gain blood parasite infections over their lifetime, but do not seem to suffer. *Journal of Ornithology*, *153*(3), 907-912.
60. van Rooyen, J., Jenkins, T., Lahlah, N., & Christe, P. (2014). North‐African house martins endure greater haemosporidian infection than their European counterparts. *Journal of Avian Biology*, *45*(5), 450-456.
61. Kim, K. S., Tsuda, Y., & Yamada, A. (2009). Bloodmeal identification and detection of avian malaria parasite from mosquitoes (Diptera: Culicidae) inhabiting coastal areas of Tokyo Bay, Japan. *Journal of Medical Entomology*, *46*(5), 1230-1234.
62. Bonneaud, C., Pérez-Tris, J., Federici, P., Chastel, O., & Sorci, G. (2006). Major histocompatibility alleles associated with local resistance to malaria in a passerine. *Evolution*, *60*(2), 383-389.
63. Loiseau, C., Zoorob, R., Garnier, S., Birard, J., Federici, P., Julliard, R., & Sorci, G. (2008). Antagonistic effects of a Mhc class I allele on malaria‐infected house sparrows. *Ecology Letters*, *11*(3), 258-265.
64. Marzal, A., Ricklefs, R.E., Valkiūnas, G., Albayrak, T., Arriero, E., Bonneaud, C., Czirják, G.A., Ewen, J., Hellgren, O., Hořáková, D. & Iezhova, T.A. (2011).

Diversity, loss, and gain of malaria parasites in a globally invasive bird. *PloS One*, *6*(7), p.e21905.

65. Gutiérrez-López, R., Martínez-de la Puente, J., Gangoso, L., Yan, J., Soriguer, R. C., & Figuerola, J. (2016). Do mosquitoes transmit the avian malaria-like parasite Haemoproteus? An experimental test of vector competence using mosquito saliva. *Parasites & Vectors*, *9*(1), 609.
66. Pérez‐Tris, J., & Bensch, S. (2005). Dispersal increases local transmission of avian malarial parasites. *Ecology Letters*, *8*(8), 838-845.
67. Knowles, S. C. L., Palinauskas, V., & Sheldon, B. C. (2010). Chronic malaria infections increase family inequalities and reduce parental fitness: experimental evidence from a wild bird population. *Journal of Evolutionary Biology*, *23*(3), 557-569.
68. Ferrer, E. S., García-Navas, V., Sanz, J. J., & Ortego, J. (2012). Molecular characterization of avian malaria parasites in three Mediterranean blue tit (*Cyanistes caeruleus*) populations. *Parasitology Research*, *111*(5), 2137-2142.
69. Podmokła, E., Dubiec, A., Drobniak, S. M., Arct, A., Gustafsson, L., & Cichoń, M. (2014). Avian malaria is associated with increased reproductive investment in the blue tit. *Journal of Avian Biology*, *45*(3), 219-224.
70. Podmokła, E., Dubiec, A., Drobniak, S. M., Arct, A., Gustafsson, L., & Cichoń, M. (2014). Determinants of prevalence and intensity of infection with malaria parasites in the Blue Tit. *Journal of Ornithology*, *155*(3), 721-727.
71. Van Rooyen, J., Lalubin, F., Glaizot, O., & Christe, P. (2013). Avian haemosporidian persistence and co-infection in great tits at the individual level. *Malaria Journal*, *12*(1), 40.
72. Dubiec, A., Podmokła, E., Zagalska-Neubauer, M., Drobniak, S. M., Arct, A., Gustafsson, L., & Cichoń, M. (2016). Differential prevalence and diversity of haemosporidian parasites in two sympatric closely related non-migratory passerines. *Parasitology*, *143*(10), 1320-1329.
73. Loiseau, C., Harrigan, R. J., Robert, A., Bowie, R. C., Thomassen, H. A., Smith, T. B., & Sehgal, R. N. (2012). Host and habitat specialization of avian malaria in Africa. *Molecular Ecology*, *21*(2), 431-441.
74. Zehtindjiev, P., Ilieva, M., Westerdahl, H., Hansson, B., Valkiūnas, G., & Bensch, S. (2008). Dynamics of parasitemia of malaria parasites in a naturally and

experimentally infected migratory songbird, the great reed warbler *Acrocephalus arundinaceus*. *Experimental Parasitology*, *119*(1), 99-110.

75. Pérez-Rodríguez, A., de la Hera, I., Bensch, S., & Pérez-Tris, J. (2015). Evolution of seasonal transmission patterns in avian blood-borne parasites. *International Journal for Parasitology*, *45*(9-10), 605-611.
76. Hellgren, O., Wood, M. J., Waldenström, J., Hasselquist, D., Ottosson, U., Stervander, M., & Bensch, S. (2013). Circannual variation in blood parasitism in a sub‐Saharan migrant passerine bird, the garden warbler. *Journal of Evolutionary Biology*, *26*(5), 1047-1059.
77. Svoboda, A., Marthinsen, G., Pavel, V., Chutný, B., Turčoková, L., Lifjeld, J. T., & Johnsen, A. (2015). Blood parasite prevalence in the Bluethroat is associated with subspecies and breeding habitat. *Journal of Ornithology*, *156*(2), 371-380.
78. Ferraguti, M., Martínez-de la Puente, J., Ruiz, S., Soriguer, R., & Figuerola, J. (2013). On the study of the transmission networks of blood parasites from SW Spain: diversity of avian haemosporidians in the biting midge *Culicoides circumscriptus* and wild birds. *Parasites & Vectors*, *6*(1), 208.
79. Zagalska-Neubauer, M., & Bensch, S. (2016). High prevalence of *Leucocytozoon* parasites in fresh water breeding gulls. *Journal of Ornithology*, *157*(2), 525-532.
80. Seimon, T.A., Gilbert, M., Neabore, S., Hollinger, C., Tomaszewicz, A., Newton, A., Chang, T. & McAloose, D. (2016). Avian Hemosporidian Parasite Lineages in Four Species of Free-ranging Migratory Waterbirds from Mongolia, 2008. *Journal of Wildlife Diseases*, *52*(3), pp.682-687.
81. Pardal, S., Alves, J.A., Zé-Zé, L., Osório, H., Rocha, A., Lopes, R.J., Potts, P., Amaro, F., Santiago-Quesada, F., Sanchez-Guzman, J.M. & Masero, J. (2014). Shorebird low spillover risk of mosquito-borne pathogens on Iberian wetlands. *Journal of Ornithology*, *155*(2), pp.549-554.
82. Ejiri, H., Sato, Y., Kim, K.S., Hara, T., Tsuda, Y., Imura, T., Murata, K. & Yukawa, M. (2011). Entomological study on transmission of avian malaria parasites in a zoological garden in Japan: bloodmeal identification and detection of avian malaria parasite DNA from blood-fed mosquitoes. *Journal of Medical Entomology*, *48*(3), pp.600-607.
83. Scaglione, F. E., Pregel, P., Cannizzo, F. T., Pérez-Rodríguez, A. D., Ferroglio, E., & Bollo, E. (2015). Prevalence of new and known species of haemoparasites in feral pigeons in northwest Italy. *Malaria Journal*, *14*(1), 99.

84. Svobodová, J., Gabrielová, B., Synek, P., Marsik, P., Vaněk, T., Albrecht, T., & Vinkler, M. (2013). The health signalling of ornamental traits in the Grey Partridge (*Perdix perdix*). *Journal of Ornithology*, *154*(3), 717-725.

85. Waldenström, J., Bensch, S., Kiboi, S., Hasselquist, D., & Ottosson, U. (2002). Cross‐species infection of blood parasites between resident and migratory songbirds in Africa. *Molecular Ecology*, *11*(8), 1545-1554.

86. Marzal, A., García-Longoria, L., Callirgos, J. M. C., & Sehgal, R. N. (2015). Invasive avian malaria as an emerging parasitic disease in native birds of Peru. *Biological Invasions*, *17*(1), 39-45.

87. Beadell, J.S., Ishtiaq, F., Covas, R., Melo, M., Warren, B.H., Atkinson, C.T., Bensch, S., Graves, G.R., Jhala, Y.V., Peirce, M.A. & Rahmani, A.R. (2006). Global phylogeographic limits of Hawaii's avian malaria. *Proceedings of the Royal Society of London B: Biological Sciences*, *273*(1604), pp.2935-2944.

88. Palinauskas, V., Valkiūnas, G., Križanauskienė, A., Bensch, S., & Bolshakov, C. V. (2009). Plasmodium relictum (lineage P-SGS1): further observation of effects on experimentally infected passeriform birds, with remarks on treatment with Malarone™. *Experimental Parasitology*, *123*(2), 134-139.

89. Palinauskas, V., Valkiūnas, G., Bolshakov, C. V., & Bensch, S. (2011). *Plasmodium relictum* (lineage SGS1) and *Plasmodium ashfordi* (lineage GRW2): the effects of the co-infection on experimentally infected passerine birds. *Experimental Parasitology*, *127*(2), 527-533.

90. Synek, P., Albrecht, T., Vinkler, M., Schnitzer, J., Votýpka, J., & Munclinger, P. (2013). Haemosporidian parasites of a European passerine wintering in South Asia: diversity, mixed infections and effect on host condition. *Parasitology Research*, *112*(4), 1667-1677.

91. Palinauskas, V., Kosarev, V., Shapoval, A., Bensch, S., & Valkiunas, G. (2007). Comparison of mitochondrial cytochrome b lineages and morphospecies of two avian malaria parasites of the subgenera Haemamoeba and Giovannolaia (Haemosporida: Plasmodiidae). *Zootaxa*, (1626), 39-50.

92. Ewen, J.G., Bensch, S., Blackburn, T.M., Bonneaud, C., Brown, R., Cassey, P., Clarke, R.H. & Pérez‐Tris, J. (2012). Establishment of exotic parasites: the origins and characteristics of an avian malaria community in an isolated island avifauna. *Ecology Letters*, *15*(10), pp.1112-1119.

93. Huang, X., Dong, L., Zhang, C., & Zhang, Y. (2015). Genetic diversity, temporal dynamics, and host specificity in blood parasites of passerines in north China. *Parasitology Research*, *114*(12), 4513-4520.
94. Cellier-Holzem, E., Esparza-Salas, R., Garnier, S., & Sorci, G. (2010). Effect of repeated exposure to *Plasmodium relictum* (lineage SGS1) on infection dynamics in domestic canaries. *International Journal for Parasitology*, *40*(12), 1447-1453.
95. Okanga, S., Cumming, G. S., Hockey, P. A., Nupen, L., & Peters, J. L. (2014). Host specificity and co-speciation in avian haemosporidia in the Western Cape, South Africa. *PLoS One*, *9*(2), e86382.
96. Dimitrov, D., Valkiūnas, G.E., Zehtindjiev, P., Ilieva, M. & Bensch, S. (2013). Molecular characterization of haemosporidian parasites (Haemosporida) in yellow wagtail (*Motacilla flava*), with description of in vitro ookinetes of *Haemoproteus motacillae*. *Zootaxa*. 3666(3), 369-81.
97. Szöllősi, E., Rosivall, B., Hasselquist, D., & Török, J. (2009). The effect of parental quality and malaria infection on nestling performance in the collared flycatcher (*Ficedula albicollis*). *Journal of Ornithology*, *150*(3), 519-527.
98. Wiersch, S. C., Lubjuhn, T., Maier, W. A., & Kampen, H. (2007). Haemosporidian infection in passerine birds from Lower Saxony. *Journal of Ornithology*, *148*(1), 17-24.
99. Stjernman, M., Råberg, L., & Nilsson, J. Å. (2008). Maximum host survival at intermediate parasite infection intensities. *Plos One*, *3*(6), e2463.
100. Martínez, J., Martínez-De La Puente, J., Herrero, J., Del Cerro, S., Lobato, E., Rivero-de Aguilar, J., Vásquez, R.A. & Merino, S. (2009). A restriction site to differentiate *Plasmodium* and *Haemoproteus* infections in birds: on the inefficiency of general primers for detection of mixed infections. *Parasitology*, *136*(7), pp.713-722.
101. Kim, K. S., Tsuda, Y., Sasaki, T., Kobayashi, M., & Hirota, Y. (2009). Mosquito blood-meal analysis for avian malaria study in wild bird communities: laboratory verification and application to *Culex sasai* (Diptera: Culicidae) collected in Tokyo, Japan. *Parasitology research*, *105*(5), 1351.
102. Glaizot, O., Fumagalli, L., Iritano, K., Lalubin, F., Van Rooyen, J., & Christe, P. (2012). High prevalence and lineage diversity of avian malaria in wild

populations of great tits (*Parus major*) and mosquitoes (*Culex pipiens*). *PLoS One*, *7*(4), e34964.

103. Isaksson, C., Sepil, I., Baramidze, V., & Sheldon, B. C. (2013). Explaining variance of avian malaria infection in the wild: the importance of host density, habitat, individual life-history and oxidative stress. *BMC Ecology*, *13*(1), 15.
104. Ventim, R., Mendes, L., Ramos, J. A., Cardoso, H., & Pérez-Tris, J. (2012). Local haemoparasites in introduced wetland passerines. *Journal of Ornithology*, *153*(4), 1253-1259.
105. Inumaru, M., Murata, K., & Sato, Y. (2017). Prevalence of avian Haemosporidia among injured wild birds in Tokyo and environs, Japan. *International Journal for Parasitology: Parasites and Wildlife*, *6*(3), 299-309.
106. Bensch, S., Waldenström, J., Jonzén, N., Westerdahl, H., Hansson, B., Sejberg, D., & Hasselquist, D. (2007). Temporal dynamics and diversity of avian malaria parasites in a single host species. *Journal of Animal Ecology*, *76*(1), 112-122.
107. Palinauskas, V., Valkiūnas, G., Bolshakov, C. V., & Bensch, S. (2008). *Plasmodium relictum* (lineage P-SGS1): effects on experimentally infected passerine birds. *Experimental Parasitology*, *120*(4), 372-380.
108. Santiago-Alarcon, D., Bloch, R., Rolshausen, G., Schaefer, H. M., & Segelbacher, G. (2011). Prevalence, diversity, and interaction patterns of avian haemosporidians in a four-year study of blackcaps in a migratory divide. *Parasitology*, *138*(7), 824-835.
109. Quillfeldt, P., Martínez, J., Hennicke, J., Ludynia, K., Gladbach, A., Masello, J.F., Riou, S. & Merino, S. (2010). Hemosporidian blood parasites in seabirds—a comparative genetic study of species from Antarctic to tropical habitats. *Naturwissenschaften*, *97*(9), 809-817.
110. Loiseau, C., Zoorob, R., Robert, A., Chastel, O., Julliard, R., & Sorci, G. (2011). *Plasmodium relictum* infection and MHC diversity in the house sparrow (*Passer domesticus*). *Proceedings of the Royal Society of London B: Biological Sciences*, *278*(1709), 1264-1272.
111. Schmid, S., Dinkel, A., Mackenstedt, U., Tantely, M. L., Randrianambinintsoa, F. J., Boyer, S., & Woog, F. (2017). Avian malaria on Madagascar: bird hosts and putative vector mosquitoes of different *Plasmodium* lineages. *Parasites & vectors*, *10*(1), 6.

112. Peev, S., Zehtindjiev, P., Ilieva, M., Träff, J., Briedis, M., & Adamík, P. (2016). Haemosporidian blood parasite diversity and prevalence in the semi-collared flycatcher (*Ficedula semitorquata*) from the eastern Balkans. *Parasitology international*, *65*(6), 613-617.
113. Lauron, E. J., Loiseau, C., Bowie, R. C., Spicer, G. S., Smith, T. B., Melo, M., & Sehgal, R. N. (2015). Coevolutionary patterns and diversification of avian malaria parasites in African sunbirds (Family Nectariniidae). *Parasitology*, *142*(5), 635-647.
114. Chasar, A., Loiseau, C., ValkiŪNas, G., Iezhova, T., Smith, T. B., & Sehgal, R. N. (2009). Prevalence and diversity patterns of avian blood parasites in degraded African rainforest habitats. *Molecular Ecology*, *18*(19), 4121-4133.
115. Bonneaud, C., Sepil, I., Milá, B., Buermann, W., Pollinger, J., Sehgal, R.N., Valkiūnas, G., Iezhova, T.A., Saatchi, S. & Smith, T.B. (2009). The prevalence of avian Plasmodium is higher in undisturbed tropical forests of Cameroon. *Journal of Tropical Ecology*, *25*(4), pp.439-447.
116. Loiseau, C., Iezhova, T., Valkiūnas, G., Chasar, A., Hutchinson, A., Buermann, W., Smith, T.B. & Sehgal, R.N. (2010). Spatial variation of haemosporidian parasite infection in African rainforest bird species. *Journal of Parasitology*, *96*(1), 21-29.
117. Howe, L., Castro, I. C., Schoener, E. R., Hunter, S., Barraclough, R. K., & Alley, M. R. (2012). Malaria parasites (*Plasmodium* spp.) infecting introduced, native and endemic New Zealand birds. *Parasitology Research*, *110*(2), 913-923.
118. Hellgren, O., Križanauskienė, A., Hasselquist, D., & Bensch, S. (2011). Low haemosporidian diversity and one key-host species in a bird malaria community on a mid-Atlantic island (São Miguel, Azores). *Journal of Wildlife Diseases*, *47*(4), 849-859.
119. Baillie, S. M., & Brunton, D. H. (2011). Diversity, distribution and biogeographical origins of *Plasmodium* parasites from the New Zealand bellbird (*Anthornis melanura*). *Parasitology*, *138*(14), 1843-1851.
120. Niebuhr, C. N., Poulin, R., & Tompkins, D. M. (2016). Is avian malaria playing a role in native bird declines in New Zealand? Testing hypotheses along an elevational gradient. *PloS One*, *11*(11), e0165918.

121. Pérez‐Rodríguez, A., Ramírez, Á., Richardson, D. S., & Pérez‐Tris, J. (2013). Evolution of parasite island syndromes without long‐term host population isolation: Parasite dynamics in Macaronesian blackcaps *Sylvia atricapilla*. *Global Ecology and Biogeography*, *22*(12), 1272-1281.
122. Martinsen, E. S., Waite, J. L., & Schall, J. J. (2007). Morphologically defined subgenera of *Plasmodium* from avian hosts: test of monophyly by phylogenetic analysis of two mitochondrial genes. *Parasitology*, *134*(4), 483-490.
